# Determination of Allergen Levels, Isoforms, and Their Hydroxyproline Modifications Among Peanut Genotypes by Mass Spectrometry

**DOI:** 10.3389/falgy.2022.872714

**Published:** 2022-05-24

**Authors:** Justin T. Marsh, Lee K. Palmer, Stef J. Koppelman, Philip E. Johnson

**Affiliations:** Food Allergy Research and Resource Program, Department of Food Science and Technology, University of Nebraska-Lincoln, Lincoln, NE, United States

**Keywords:** *Arachis hypogea*, Ara h 1, Ara h 2, Ara h 3, Ara h 6, food allergy, LC-MS/MS, post-translational modification

## Abstract

The recently published reference genome of peanuts enables a detailed molecular description of the allergenic proteins of the seed. We used LC-MS/MS to investigate peanuts of different genotypes to assess variability and to better describe naturally occurring allergens and isoforms. Using relative quantification by mass spectrometry, minor variation of some allergenic proteins was observed, but total levels of Ara h 1, 2, 3, and 6 were relatively consistent among 20 genotypes. Previously published RP-HPLC methodology was used for comparison. The abundance of three Ara h 3 isoforms were variable among the genotypes and contributed to a large proportion of total Ara h 3 where present. Previously unpublished hydroxyproline sites were identified in Ara h 1 and 3. Hydroxylation did not vary significantly where sites were present. Peanut allergen composition was largely stable, with only some isoforms displaying differences between genotypes. The resulting differences in allergenicity are of unknown clinical significance but are likely to be minor. The data presented herein allow for the design of targeted MS methodology to allow the quantitation and therefore control of peanut allergens of clinical relevance and observed variability.

## Introduction

Peanuts contain multiple allergens, which differ in abundance (1). Furthermore, each allergen family is a mixture of isoforms, with the tetraploidy of the peanut producing more complexity (2). One way of assessing the peanut allergen profile has been the use of reverse-phase high-pressure liquid chromatography (RP-HPLC) (3). Each UV-absorbing peak is typically assigned to a particular allergen protein family. Assessment of extracts by this method is dependent on the quality and robustness of the method and the evidence supporting the assignments of the RP-HPLC peaks. Supporting evidence for allergen identification has included antibody-based detection and classical biochemical methods such as sodium dodecyl sulfate-polyacrylamide gel electrophoresis (SDS-PAGE). Similar studies of the allergen profiles of peanut genotypes have been performed by Pandey et al. and Wu et al. focusing on analysis by ELISA and SDS-PAGE densitometry, respectively, where both studies showed that genotypes do contribute a level of variability to the present profile of allergens (4, 5).

The most abundant allergens of peanut seed are the 2S albumins (Ara h 2 and 6) and the cupins (Ara h 1 and 3) (1), where Ara h 2 has been regarded as the most important effector cell responses and diagnosis of peanut allergy (6). Less abundant allergens of the peanut seed include the profilin (Ara h 5), a 2S albumin (Ara h 7), the non-specific lipid-transfer proteins (nsLTP) (Ara h 9, 16, and 17), the defensins (Ara h 12 and 13), the Bet v 1-type protein (Ara h 8), the oleosins (Ara h 10, 11, 14, and 15) (1), and cyclophilin (Ara h 18) (7). Post-translational modifications may also play a significant role in allergenicity, as Bernard et al. evaluated that hydroxyprolination (HyP) of Ara h 2 was shown to be important in the elicitation of an immune response (8).

Mass spectrometry (MS) using data-dependent workflows with label-free quantitation was leveraged to determine the fine composition of allergenic proteins (allergen family, isoform, and post-translational modifications thereof) of the peanut seed. These workflows were compared to RP-HPLC analysis to examine the relative utility and to validate the use of RP-HPLC as a tool to monitor peanut extract batch-to-batch variation in terms of the allergen content. Peanut extracts representing 20 different genotypes (across 4 market types) previously characterized by RP-HPLC were evaluated using MS (3). Recent descriptions of the peanut genome allowed detailed interpretation of proteomic data (2). In addition to determining the utility of MS methodology for detailed studies of allergen comparability, the data obtained also allowed us to identify the most variable of the peanut allergens.

## Materials and Methods

### Chemicals

All reagents for protein preparation were of analytical reagent grade. All reagents for MS sample preparation and analyses were of MS grade. Trifluoroacetic acid and iodoacetamide (IAA) were obtained from Sigma (St. Louis, MO, USA). Tryptic digested rabbit glycogen phosphorylase B (P00489.3) was obtained from Waters (Manchester, UK). Pierce trypsin (MS grade) and Pierce C18 spin columns were obtained from Thermo Scientific (Waltham, MA, USA). Dithiothreitol (DTT) was obtained from Arcos Organics (Geel, Belgium). Ammonium bicarbonate was obtained from Honeywell Fluka (Charlotte, NC, USA). Methanol, acetonitrile (ACN), water, and formic acid (all Optima LC/MS grade) were obtained from Fisher (Hampton, NH, USA).

### Sample Extraction for RP-HPLC and Liquid Chromatography-Mass Spectrometry LC-MS/MS

The same extracts of raw peanut protein from 20 different peanut genotypes representing 4 market types were used as described in Koppelman et al. (3). Briefly, extracts were generated from a 100 mg ml^−1^ peanut paste extraction in 10 mM ammonium bicarbonate, pH 7.9, which were subsequently clarified a total of three times. RP-HPLC was performed, and Ara h 1, 2, 3, and 6 were quantified relative to external calibration curves of purified allergens. A table of samples is listed in Supplementary Table S1 with associated protein concentrations determined previously.

### LC-MS/MS—Reduction, Alkylation, and Digestion

Digestions were performed in duplicate in the following manner. Extract volumes corresponding to 10 μg were diluted in 50 mM ammonium bicarbonate to a volume of 15 μl. Samples were reduced by adding 10.5 μl of water and 1.5 μl of 100 mM dithiothreitol and incubating at 95°C for 5 min. The samples were alkylated by adding 3 μl of 100 mM of iodoacetamide and incubating at room temperature in the dark for 20 min. The sample was digested by adding 1 μl of 100 ng μl^−1^ Pierce trypsin protease, MS grade, and incubated at 37°C for 3 h. A further 1 μl of 100 ng μl^−1^ trypsin was added and samples were incubated at 30°C overnight. The digested sample was applied and eluted from a Pierce C18 spin column according to the instructions of the manufacturer. Samples were frozen at −80°C prior to lyophilization by centrifugal evaporation, and the dried samples were resuspended in a mixture of 45 μl of 5% ACN/0.1% formic acid and 5 μl of 200 fmol μl^−1^ rabbit glycogen phosphorylase (Waters, Manchester, UK).

### LC-MS/MS—Detailed Parameters

One-dimensional microscale liquid chromatography separation of tryptic peptides (5-μl injections equivalent to 1,000 ng and 100 fmol glycogen phosphorylase on column) was performed in duplicate (2 technical replicates) with an UltiMate 3000 RSL® liquid chromatography system (Thermo Scientific™) equipped with a Javelin™ Direct-Connection Column Filter, 2.1 mm (Thermo Scientific™), a Hypersil Gold aQ C18 1.9 μm, 20 x 2.1 mm pre-column (Thermo Scientific™), and a Hypersil Gold C18 1.9 μm, 100 x 1 mm analytical reverse-phase column (Thermo Scientific™). Mobile phase A consisted of water containing 0.1% (v/v) formic acid, whereas mobile phase B was 100% (v/v) ACN containing 0.1% (v/v) formic acid. The sample was injected and peptides were eluted using a gradient of 2–40% mobile phase B over 60 min at a flow rate of 60 μl min^−1^. The analytical column temperature was maintained at 35°C.

The mass spectrometric analysis utilized a Q-Exactive Plus™ Hybrid Quadrupole-Orbitrap™ MS (Thermo Scientific™) in the data-dependent mode with survey scans acquired at a resolution of 70,000 at m/z 400, whereas the target value for the fragment ion spectra was set to the resolution of 17,500 at m/z 400. Up to the top 10 most abundant isotope patterns with charges 2 to 4 from the survey scan were selected with an isolation window of 1.5 Thomsons and fragmented by higher-energy collisional dissociation with normalized collision energies of 27. The maximum ion injection times for the survey scan and the MS/MS scans were 100 and 60 ms, respectively, and the ion target value for scan modes was set to 1E6 and 2E5, respectively. Repeat sequencing of peptides was kept to a minimum by the dynamic exclusion of the sequenced peptides for 10 s.

### Analysis of LC-MS/MS Data

PEAKS version 8.5 software was used to process all data-dependent acquisition mass-spectral data (9). Protein identifications were obtained by searching a database of peanut allergens derived from www.peanutbase.org and listed in Marsh et al. Supplementary S11 (10) including the sequence for rabbit glycogen phosphorylase B (P00489.3) (total sequences: 120). The following data analysis parameters were used: no mis-cleavages, carbamidomethylation as a fixed modification, oxidation of methionine and proline as a variable modification, parent mass error tolerance of 2 ppm, fragment mass error tolerance of 0.02 Da, false discovery rate of 1 %, and charge states between 2 and 4 were accepted. Protein quantities (average peak area of top3 MS1) were normalized according to the known amount of rabbit glycogen phosphorylase B spiked into the sample (100 fmol on column). Data were analyzed using Excel 2013 (Microsoft) and Prism version 9.0 (GraphPad software). The proteomic data have been deposited to the PRIDE Archive (http://www.ebi.ac.uk/pride/archive) via the PRIDE partner repository with the dataset identifier PXD028086.

For allergen quantitation, two strategies were employed (shared and unique). In shared quantitation, the peptides with the largest parent ion abundance of those peptides present in the greatest number of isoforms were manually chosen. In unique quantitation, the peptides with the largest parent ion abundance of those peptides unique to one isoform were manually chosen. If an allergen family required more than one group of “shared” peptides, each group was summed to produce the total allergen abundance.

Isoform nomenclature is presented in Supplementary Table S2, where proteins were grouped based on homology. Supplementary Tables S3A-E show the identity matrices of WHO/IUIS peanut allergens against the isoform nomenclature presented herein. For both strategies, allergens were assumed to form the bulk of protein in the peanut seed. Allergen quantities are presented as mean protein content % [weight (allergen) /weight (total allergen)] in peanut for each allergen family and the standard error of the mean, as determined by the ratio of an allergen (g)/total allergen (g). Allergen (g) was determined by converting normalized molar values per allergen according to assumed molecular weights (Da) (Ara h 1: 61,900; Ara h 2: 17,300; Ara h 3: 65,000; Ara h 6: 14,900; Ara h 7: 17,300; Ara h 8: 16,500; Ara h 9: 13,000; Ara h 10: 18,000; and Ara h 11: 14,000). Total allergen (g) was found by summing all determined allergen weights. Supplementary Figure S2 outlines the strategy.

Proteins were segregated based on abundance into high abundance (>2.5%), mid abundance (2.5–0.5%), and low abundance (<0.5%). For the LC-MS/MS vs. RP-HPLC comparison, Ara h 1, 2, 3, and 6 were summed, as the RP-HPLC data only reported for these allergens (3). A supplementary excel file is also supplied giving the intensities and normalized concentrations of the selected peptides.

Presumptive HyP sites on high-abundance allergens (Ara h 1, 2, 3, and 6) were reported if the relative abundance of the tryptic peptide with hydroxyproline was at least 5% of the abundance when compared to the unmodified form or >2 fmol on the column, with an associated A score of >10. In the case of a peptide with multiple HyP sites, the abundances of these peptides were summed prior to comparison with the unmodified form. An overview of the experimental and data analysis methodology is shown in Supplementary Figures S1, S2. Examples of spectra are shown in Supplementary Figure S6.

We have used standard deviation (SD) and %CV to analyze the variation of the allergens, isoforms, and HyP sites across the peanut genotypes, and we use the standard error of the mean (SEM) (SD of the mean of the two biological replicates / √(n)) when we report within the genotype.

## Results

### Peanut Seed Proteome

The peanut seed proteome becomes increasingly complex moving from allergen families to the component isoforms, and accurate identifications are complicated by the breadth of sequences reported in the literature and submitted to databases without protein-level evidence. Therefore, peanut allergens were identified relative to the recently published Tifrunner genome to restrict the duplication of allergen sequences (2, 10). Supplementary Table S2 lists the protein NCBI accessions (from the peanut genome), tryptic proteome coverage, the number of total and unique peptides detected, and the allergens. The two isoforms of Ara h 1 were confirmed by multiple unique peptides. The isoforms of the allergens, Ara h 2, 6, and 7, were confirmed by only one unique peptide, due to the high identity of the two isoforms. Of the twenty Ara h 3 isoforms previously predicted (10) fourteen were confirmed by multiple unique peptide detection. The remaining six isoforms were highly similar and so could not be uniquely identified. In these cases, evidence is presented for the existence of one or both of a pair of isoforms.

Broad proteomic evaluation of peanut genotypes provided insights into the Tifrunner genome. The UniProt and NCBI databases have a far greater number of Ara h 1 genes that have been submitted but were neither identified within the Tifrunner genome nor by LC-MS/MS. Of the indiscernible pairs of Ara h 3 isoforms, Ara h 3.6 may be a pseudogene as no signal peptide was identified and the *N*-terminus was found to be truncated but as this isoform could not be discriminated from Ara h 3.3, there is no evidence to support this. The NCBI *Arachis hypogaea* Annotation Release 100 correctly identifies many of the genes, apart from Ara h 3.16, which was identified as a pseudogene due to misidentification of the start site. Neither proteomic nor genomic evidence supports the existence of Ara h 7.0301 (UniProt Q647G8) and is likely a frame-shift error in the relevant EST as suggested by Schmidt et al. (11) and Blankestijn et al. (12). As the mid- and low-abundance (2.5–0.5% (w/w) and <0.5 % (w/w), respectively) allergen families were far less prevalent in seed protein extracts, experimental confirmation of their isoforms ranged from entirely unsuccessful, where none of the eight Ara h 5 isoforms were identified, to highly successful, where eight of the Ara h 10 and 11 oleosins were identified (refer to Supplementary Table S5B).

### Quantitation

To appropriately compare RP-HPLC and LC-MS/MS, a two-fold strategy was applied where a shared peptide quantitation (peptides shared between isoforms) was used to evaluate the quantitation of the allergen families and a unique peptide quantitation (peptides present in only one isoform) was used to deconvolute the isoforms. The use of shared peptide quantitation allows the use of peptides that are not unique among isoforms to improve the robustness of the quantitation, whereas unique peptide quantitation focuses on the separation of isoforms and therefore ratios of the isoforms present. These strategies become important particularly for Ara h 3 as many highly identical isoforms would otherwise make quantitation impossible, if only unique peptides were used (Excel supplementary shows the underreporting of the top 3 unique quant vs. the top 3 shared quant). Supplementary Table S4 shows the meta-data of the overall number of peptides of the Ara h 3 family, for the shared and unique quantitation.

#### Total Allergen Quantitation by LC-MS/MS

**Figure 1:**
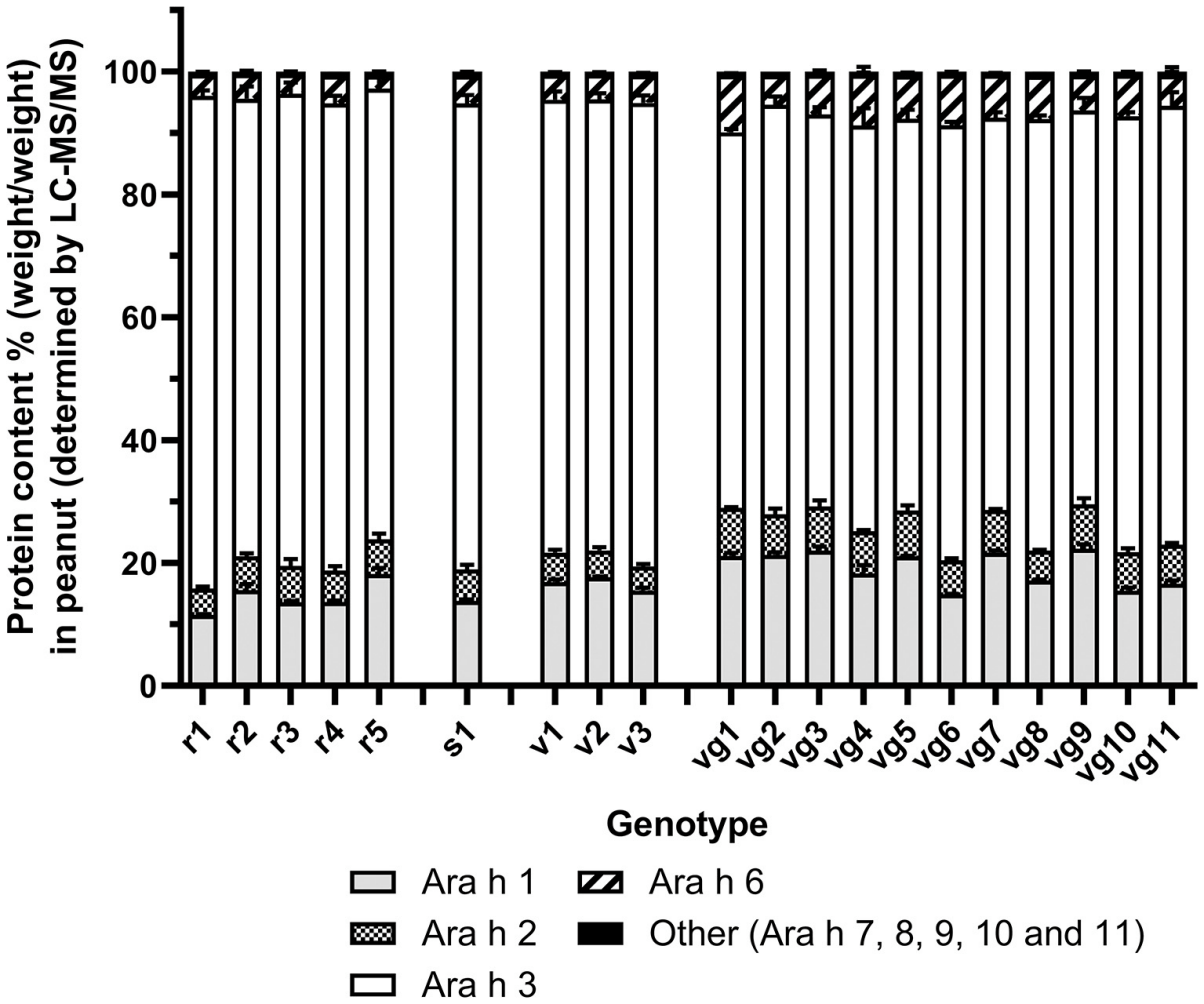
Protein quantitation of Ara h 1, 2, 3, 6, and other (Ara h 7–11) as determined by shared peptide quantitation. Trypsin-digested protein spiked with glycogen phosphorylase peptides was analyzed by LC-MS/MS. Allergens were quantified by the shared peptides (Supplementary Table S5A) and normalized to glycogen phosphorylase. Stacked bar histogram plots show the mean protein content % [weight (allergen)/weight (total allergen)] in peanut for each allergen family and standard error of the mean (bar) from two biological replicates, which had two technical replicates for each sample (left *y*-axis). Each peanut genotype analyzed is shown on the *x*-axis.

A total of 33 peptides were used to quantify the high-abundance allergen isoforms (Supplementary Table S5A). A further 16 peptides were used to quantify the mid- and low-abundance allergen isoforms (Ara h 7, 8, 9, 10, and 11; Supplementary Table S5B). Most of the proteins detected in all the peanut seed extracts were the high-abundance allergens, accounting for over 99.4 ± 0.01% of detectable allergen protein weight (Figure 1 and Table 1). The mid- and low-abundance allergens, although less abundant, were identified and quantified (Supplementary Figure S3).

**Table 1:** Overview of high-abundance peanut allergen abundancies.

**Percentage (using % (w/w) protein)**	**Ara h 1**	**Ara h 2**	**Ara h 3**	**Ara h 6**
Mean (Standard Deviation)	17.4 (3.3) %	5.9 (1.1) %	70.6 (5.4) %	5.5 (1.9) %
Range (Genotype)	11.5 (r1)-22.4 (vg9)	3.9 (v3)-7.8 (vg1)	61.2 (vg1)-80.2 (r1)	2.2 (r5)-9.3 (vg1)
Fold difference^a^	1.9	2.0	1.3	4.3

a *Fold difference determined by the max/min (refer to Range)*.

Across market types, peanuts displayed a maximum fold difference of 2 or less for Ara h 1, 2, and 3. Ara h 6 was more variable with a maximum fold difference of 4.3 (Table 1). A comparison of market types and allergens by two-way ANOVA with *post hoc* Tukey's test showed significant differences (*p* < 0.001) in Ara h 1 abundance between Runner vs. Virginia market types (14.5 vs. 19.3 % w/w) and Ara h 3 abundancies between Virginia vs. all other market types (66.6 vs. 76.2, 75.9, 74.2 % w/w). The eleven Virginia market types appeared to separate into two subgroups. Subgroup 1 (vg6, vg8, vg10, and vg11) was not significantly different from the other market types; however, subgroup 2 (vg1, vg2, vg3, vg4, vg5, vg7, and vg9) showed significant differences in Ara h 1 abundance vs. all other market types (21.2 vs. 14.5, 13.8, 16.7 % w/w) and Ara h 3 abundancies vs. all other market types (64.7 vs. 76.2, 75.9, 74.2 % w/w). The data is shown in Supplementary Figure S4.

#### Comparison of RP-HPLC and LC-MS/MS for Determination of Allergen Content

**Figure 2:**
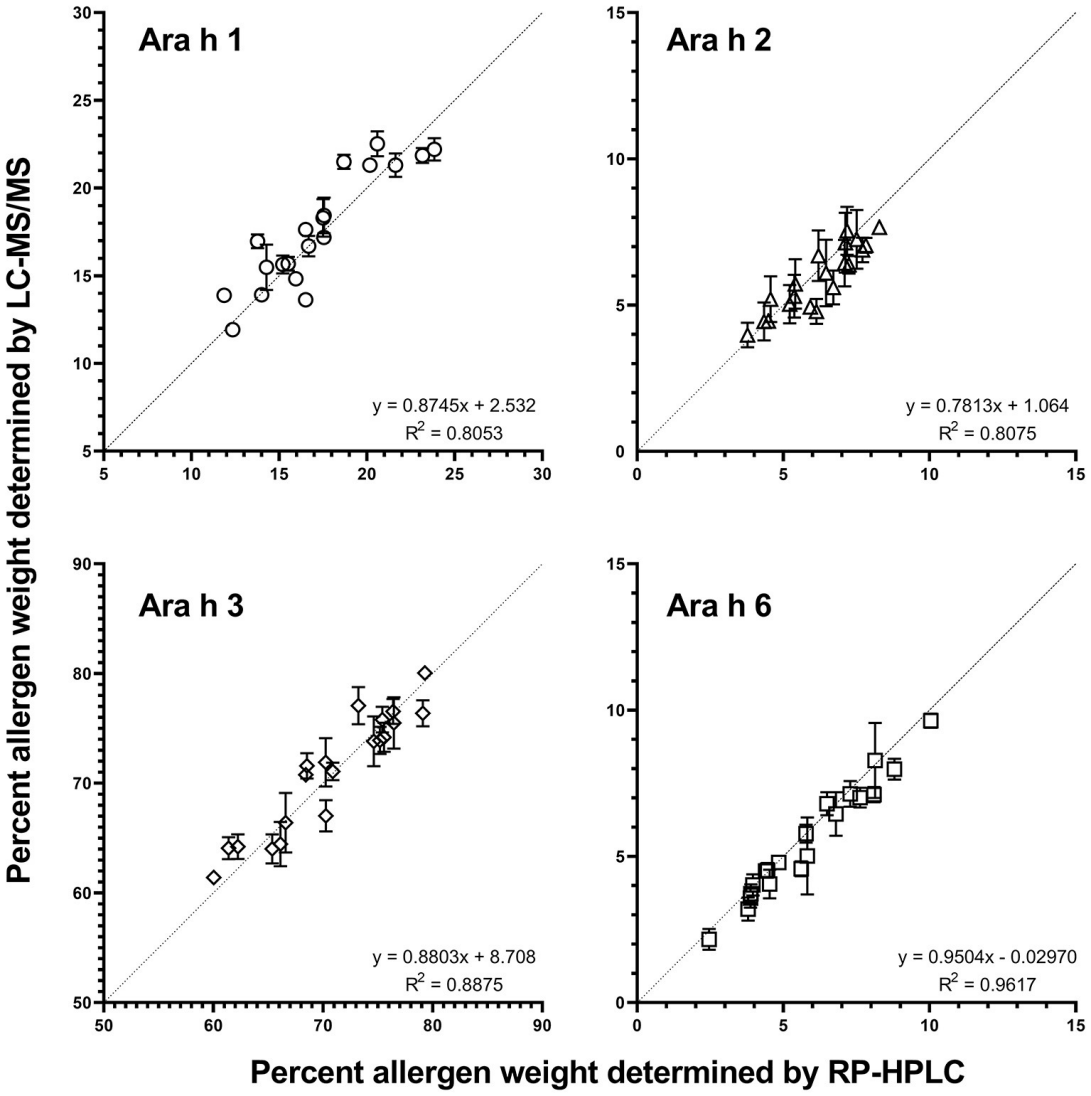
Comparison of Ara h 1, 2, 3, and 6 percentages as determined by RP-HPLC (1) vs. LC-MS/MS detection. The average percent protein weight in each genotype [allergen (g)/total protein (g)] for each allergen family as determined by RP-HPLC (*x*-axis) vs. LC-MS/MS (*y*-axis) family is shown. Each data point represents one genotype. Each measurement represents the mean and standard error of two biological replicates (each with 2 technical replicates for LC-MS/MS). The correlation coefficient (RP-HPLC vs. LC-MS/MS) for all allergens and all peanut genotypes was 0.9966 (best fit y = 1.005x−0.1193) (*p* < 0.0001 significantly non-zero slope).

The two independently generated allergen datasets determined by LC-MS/MS and RP-HPLC (3) were compared using the same extracts (Figure 2). The best fit linear regression was determined to be y = 1.005x−0.1193 (*p* < 0.0001, R^2^= 0.9966), demonstrating that the determined allergen content from both methods is very comparable and indicating that the database used represents all the high-abundance peanut allergen isoforms and that the choice of peptides used for quantitation captures the allergen content of each extract.

### Allergen Isoform Quantitation Using Peptides Unique to Allergen Isoforms

**Figure 3:**
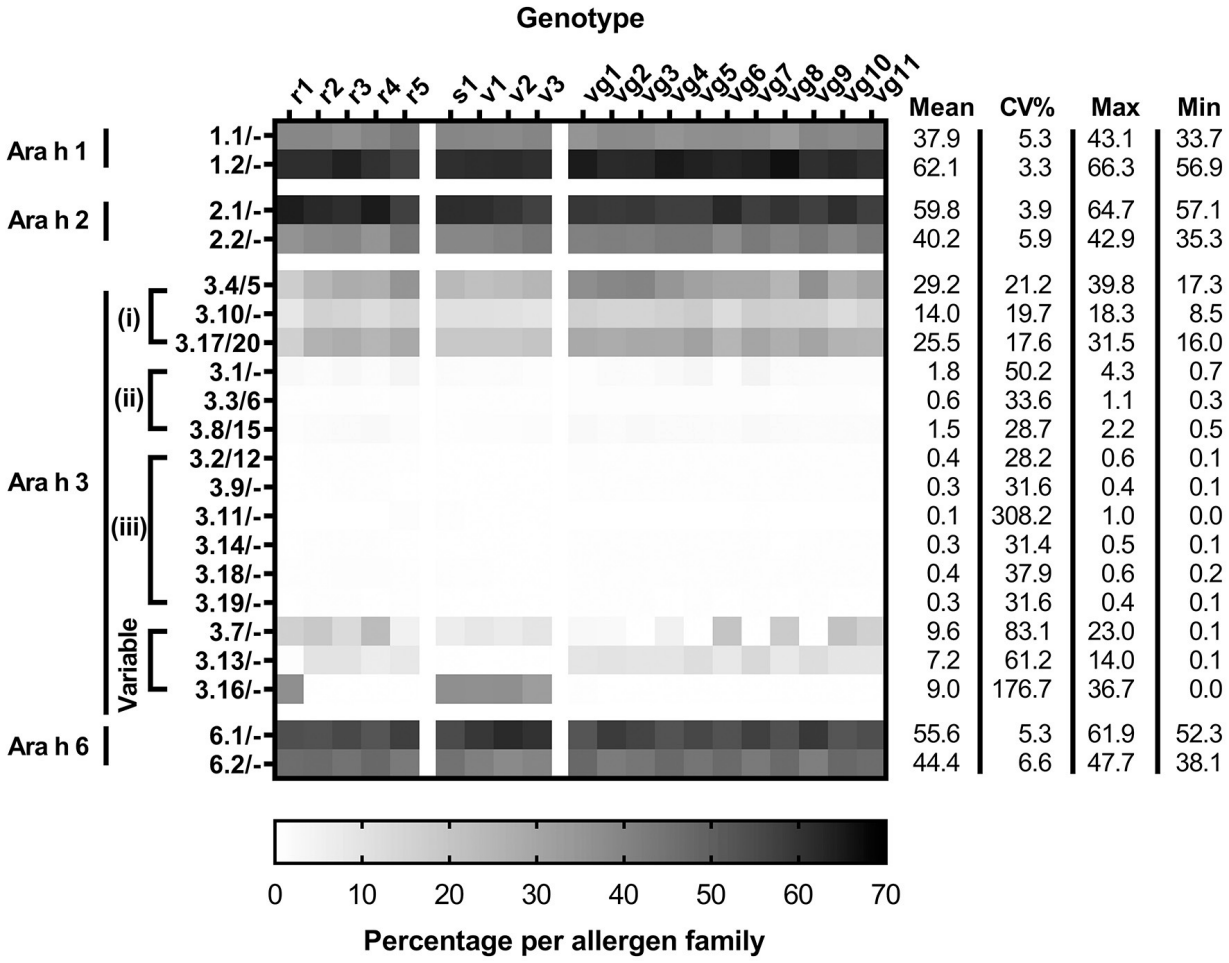
Isoform distributions of Ara h 1, 2, 3, and 6 per genotype determined by unique peptide quantitation. Allergen quantitation is expressed as the percentage of allergen protein [allergen (g)/total protein (g)] per allergen family. Subgroupings of Ara h 3 include (i) high-abundance, (ii) mid-abundance and (iii) low-abundance. Each measurement from two biological replicates, which had two technical replicates for each peanut genotype, includes the mean, percent coefficient of variation (CV%), maximum, and minimum values.

A total of 49 peptides were used to quantify the isoforms of the high-abundance allergens (Supplementary Table S2). The isoform pairs of Ara h 1, 2, and 6 were expressed at similar ratios across the genotypes (Figure 3 and Supplementary Figure S5). The most abundant isoforms detected were Ara h 1.2, Ara h 2.1, and Ara h 6.1, respectively. Quantitation of Ara h 2 and 6 isoforms using unique peptides showed substantial decreases compared to that derived from using shared peptides (2.7-folds less for Ara h 2 and 2.3-folds less for Ara h 6) (refer to supplementary excel). This is likely due to reliance on a single unique peptide to discriminate the isoforms, which were detected poorly for both allergens, resulting in an underestimation of abundance.

**Figure 4:**
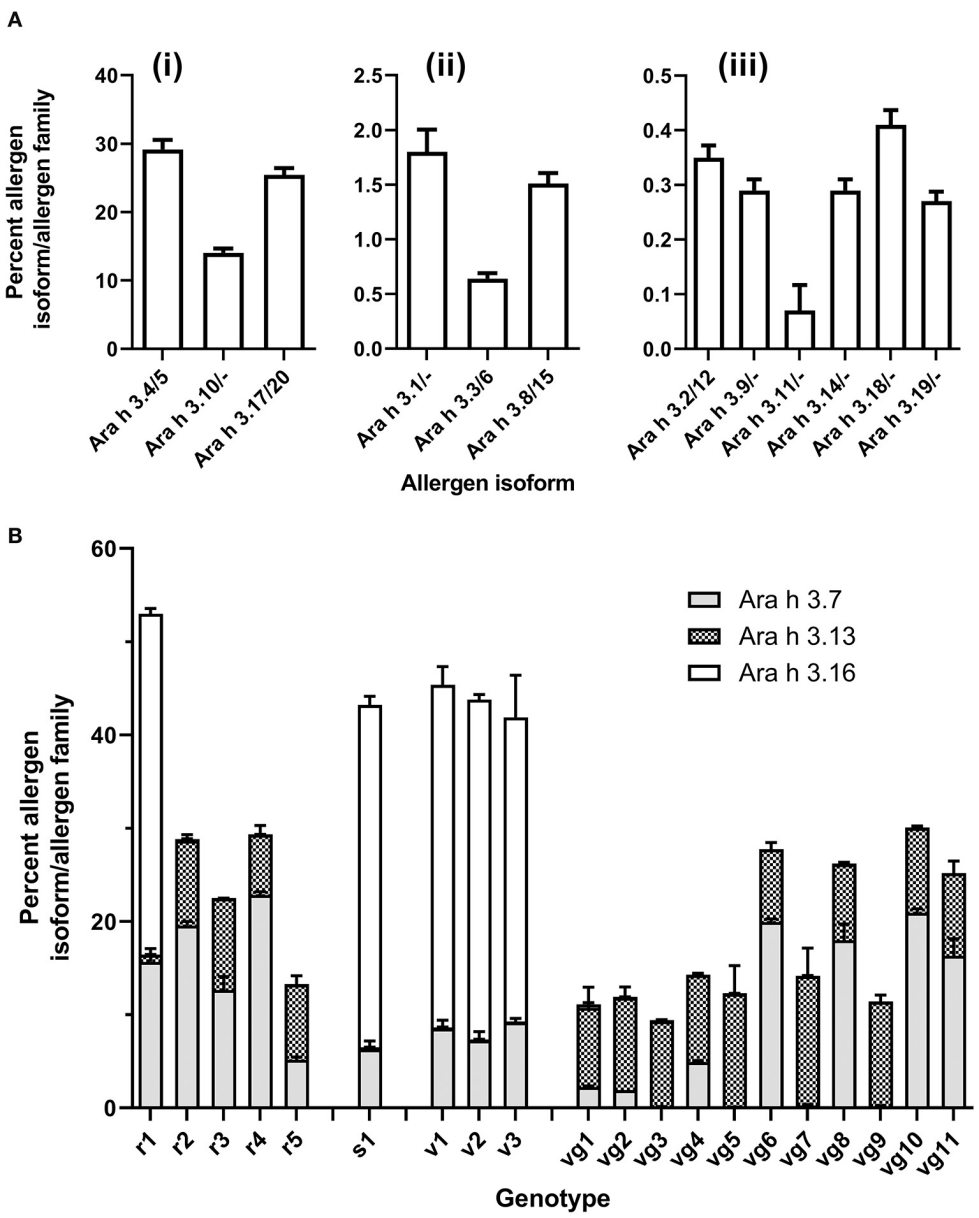
Isoform distribution of Ara h 3 across genotypes as determined by specific isoform (unique) peptide quantitation. Allergens were quantified using unique peptides (Supplementary Table S2) and normalized to glycogen phosphorylase. Stacked bar histogram plots show the mean protein content % [weight (allergen) /weight (total allergen)] in peanut for each allergen family and standard error of the mean (bar) from two biological replicates, which had two technical replicates for each sample (left *y*-axis). Each peanut genotype analyzed is shown on the *x*-axis. **(A)**, Ara h 3 isoforms, in all genotypes (i) high-abundance Ara h 3 isoforms (> 2.5 % (w/w): Ara h 3.4/5, Ara h 3.10, and Ara h 3.17/20 (ii) mid-abundance Ara h 3 isoforms [2.5–0.5% (w/w)]: Ara h 3.1, Ara h 3.3/6, and Ara h 3.8/15 (iii) low-abundance Ara h 3 isoforms (<0.5 % (w/w)): Ara h 3.2/12, Ara h 3.9, Ara h 3.11, Ara h 3.14, Ara h 3.18, and Ara h 3.19. **(B)**, Variable Ara h 3 isoforms: Ara h 3.7, Ara h 3.13, and Ara h 3.16.

The Ara h 3 gene family is more complex compared to the other high-abundance peanut allergen families, as it has multiple isoforms, many of which are nearly identical to the point that no detectable peptides can discriminate them. Where pairs of indistinguishable Ara h 3 isoforms, such as Ara h 3.4 and 3.5, exist, these are denoted as Ara h 3.4/5. The Ara h 3 isoforms were segregated into four groups based on abundance: high, mid, and low abundances, and variable. The high-abundance Ara h 3 isoforms include Ara h 3.4/5, Ara h 3.17/20, and Ara h 3.10 [Figure 4A (i)]. The mid-abundance Ara h 3 isoforms include Ara h 3.1, Ara h 3.3/6, and Ara h 3.8/15 [Figure 4A (ii)]. Seven isoforms comprise the low-abundance Ara h 3 isoforms including Ara h 3.2/12, Ara h 3.9, Ara h 3.11, Ara h 3.14, Ara h 3.18, and Ara h 3.19 [Figure 4A (iii)]. Detectable levels of 17 of the 20 Ara h 3 isoforms were comparable across all genotypes of peanut, with the other three showing marked genotype-dependent expressions (Figures 3, 4).

The isoforms Ara h 3.7, Ara h 3.16, and Ara h 3.13 had variable abundance among the genotypes (Figure 4B). When present, these variable isoforms were a large proportion of the total Ara h 3 content. The SDS-PAGE published by Koppelman et al. (3) of the same extracts utilized here evidenced a 38 kDa band exhibiting variable intensity. The appearance of this is correlated with detectable levels of the Ara h 3.7 and/or Ara h 3.16 (calculated as 37.7 kDa). This variable band may be the acidic subunit of Ara h 3.7 and/or 3.16 based on the expression pattern and observed molecular weight, but a direct analysis of this band would be required to definitively prove this. All other high-abundance and variable Ara h 3 acidic subunits, including Ara h 3.4/5/10/13/17/20, are predicted to migrate at approximately 40 kDa on SDS-PAGE (refer to Supplementary Table S4). Interestingly, Mamone et al. evaluation of the 3 different varieties of peanut (Zambia, China, and Virginia) showed that a variable molecular weight band on their SDS-PAGE gels was annotated as an Ara h 3 or Ara h 3 acidic subunit (13). Differences in observed molecular weight and band intensity of the acidic subunits of Ara h 3 have previously been attributed to post-translational proteolysis (14), which has also been observed for Ara h 2 and Ara h 6 (15, 16).

Ara h 3.7 was present in all genotypes but with widely ranging abundance from 0.07 (vg5) to 22.8% (r4). Ara h 3.13 was identified in 15 genotypes at high levels with abundances ranging from 6.49 (r4) to 13.9% (vg7). Lower levels were found in the other 5 genotypes (r1, s1, v1, v2, and v3) with abundances ranging from 0.75 (r1) to 0.06% (v3). Ara h 3.16 was identified at high abundance in 5 genotypes (r1, s1, v1, v2, and v3) with abundance ranging from 36.71 (s1/v1) to 32.61% (v3). Lower levels were found in the other 15 genotypes with abundances ranging from 0.01 (vg11) to 0.36% (vg1). Ara h 3.13 and Ara h 3.16 expression seems to be inversely correlated and is associated with market type where Spanish/Valencia genotypes highly express Ara h 3.16, whereas Virginia/Runner genotypes highly express Ara h 3.13. In this respect, the experimental Runner variety examined (r1) behaves like a Spanish/Valencia genotype. The genes for Ara h 3.13 and Ara h 3.16 reside on the same chromosome separated by over 2 million nucleotides, suggesting that the effect is not a cis-suppression effect. Gavage et al. examined various peanut genotypes using a proteomic approach but used peptides that did not entirely capture the sets of similar Ara h 3 isoforms resulting in some of the variability observed, particularly involving the variable isoforms Ara h 3.7 and Ara h 3.16 (17). Our assessment of the peptides identified by Gavage et al. indicates that their Spanish peanuts cultivated in China had greater amounts of Ara h 3.13 than Ara h 3.16, and their Virginia peanuts grown in Israel had greater amounts of Ara h 3.16 than Ara h 3.13, which is the opposite of what was observed in this data, and suggests that factors beyond market type may be associated with expression of Ara h 3.13 and Ara h 3.16. Our re-evaluation of other LC-MS/MS studies of peanut genotypes also indicates that proteomic differences observed by 2DE could be attributed in part to the variable isoforms of Ara h 3 described by this data (18, 19). Grouping the Ara h 3 isoforms similarly to the shared quantitation evidenced that two groups (Ara h 3.4/5/10/13/17/20 and Ara h 3.7/16) together comprise most of the Ara h 3 in all genotypes studied (94.5 ± 0.35%). Per group, these sets of isoforms are highly identical (>93%), but less so across groups (>73%).

### Hydroxyproline-Rich Regions of the Peanut Allergens

**Figure 5:**
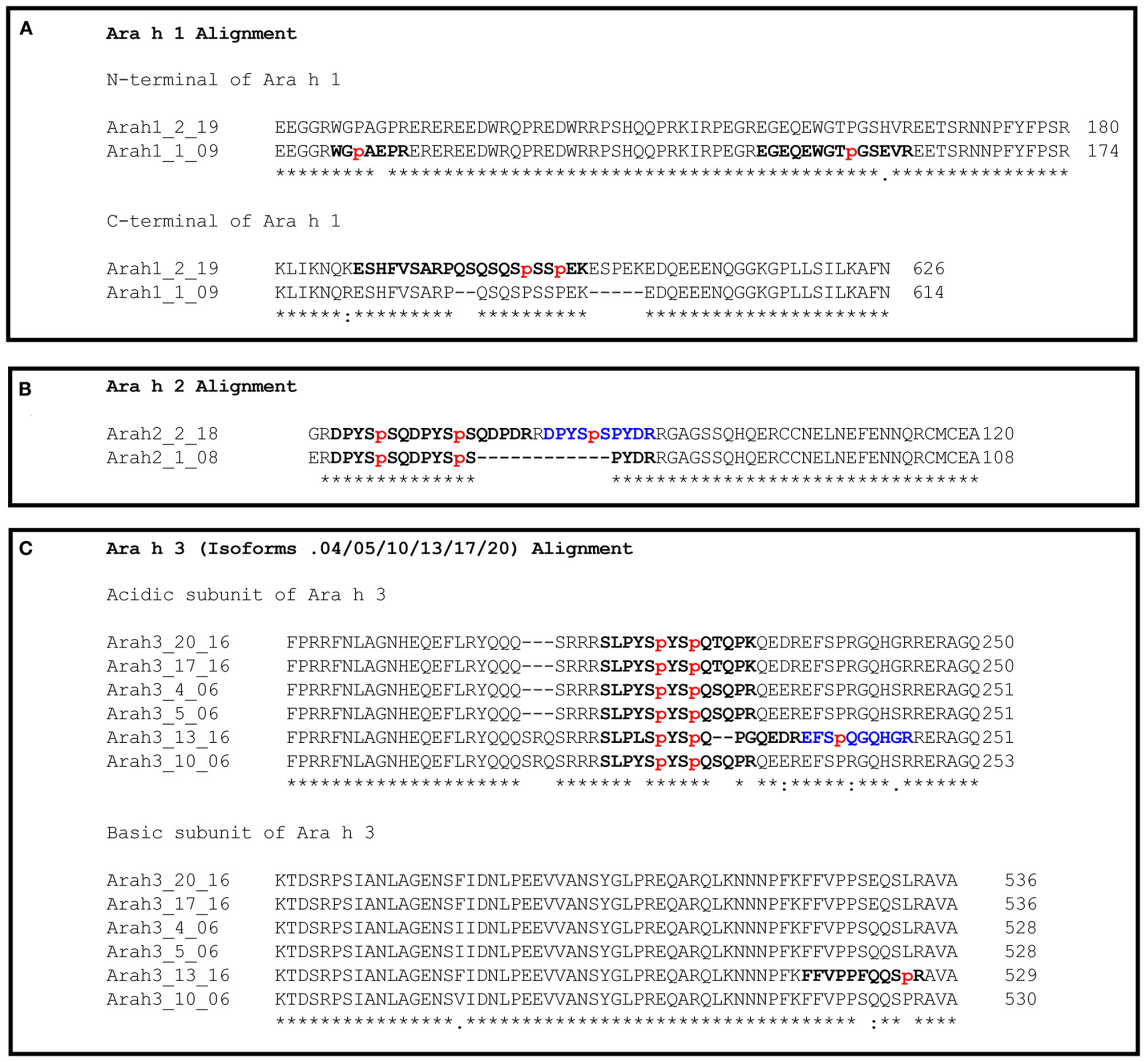
Sites of hydroxyproline-rich regions of Ara h 1, 2, and 3. The most abundant HyP-modified tryptic peptides detected are bolded. The sites of HyP are shown in lower case and colored red (refer to Supplementary Table S6). **(A)** shows the predominant sites of HyP modification in Ara h 1. **(B)** shows the predominant sites of HyP modification in Ara h 2. **(C)** shows the predominant sites of HyP modification in Ara h 3. Alignment was conducted using Clustal Omega (v1.2.4).

Post-translational hydroxylation of proline has been reported to occur in a repeat region of Ara h 2 and has demonstrated implications for IgE binding and possibly allergy (8). Our data show that HyP also occurs in other high-abundance peanut allergens and further allows comparison of peanut genotypes with respect to the occurrence and prevalence of HyP. Tryptic HyP-modified peptides were quantified relative to their corresponding non-modified form (Supplementary Table S6 and Supplementary Figure S6). Due to possible differences in the detection of HyP-modified and non-HyP-modified peptides, this should not be interpreted as a quantitative determination of the degree of hydroxylation. However, changes in the relative abundance of observed modification between peanut genotypes would allow for the determination that some genotypes differ in HyP of key allergenic proteins. Only peptides where HyP-modified peptide abundance was >2% of the unmodified peptide abundance were reported here. Figure 5 summarizes the allergen isoforms with the most prevalent HyP sites identified (Supplementary Table S6 giving the percentage). Novel HyP regions in Ara h 1 and a subset of Ara h 3 isoforms were identified, but no HyP-rich regions were detected in either Ara h 6 or the other less abundant allergens. Apostolovic et al. demonstrated that the two isoforms of Ara h 7 do contain HyP modifications, but these were identified using purified Ara h 7 rather than whole peanut extracts (20). The ratio of the peptide abundancies was remarkably consistent across the genotypes studied indicating that no genotype-dependent hydroxylation occurs and that all genotypes modify the same peptide motifs.

#### Novel Ara h 1 HyP Region

Three HyP-modified regions were identified corresponding to both Ara h 1 isoforms across all genotypes. (Figure 5A and Supplementary Figures S7A-C). Two HyP peptide sets were identified in the *N*-terminus of Ara h 1.1 at positions 111 and 155, respectively, and the last HyP region was found in the *C*-terminus of Ara h 1.2 corresponding to positions 594 and 597. Both Ara h 1.1 HyP regions were far less abundant than the non-HyP-modified, whereas the Ara h 1.2 HyP peptides were exclusively identified with some patterns of HyP modification.

#### Ara h 2 HyP Region

A single HyP-modified region was identified for both Ara h 2 isoforms across each genotype (Figure 5B and Supplementary Figures S8A-C). Three peptides were identified corresponding to Ara h 2.1 with different HyP patterns among positions 67, 74, and 76, and the most predominant was modified at both positions 64 and 74. No peptides without HyP were identified for this region. Four peptides were mapped to two regions of Ara h 2.2, the first exclusively identified with HyP at positions 67 and 74, and the second predominantly identified with HyP at position 86. The most abundant HyP sites identified by LC-MS/MS were also identified by Bernard et al. (8), although less abundant HyP motifs complimented the most abundant motifs identified suggesting that most, if not all Ara h 2 protein has at least one HyP modification.

#### Novel Ara h 3 HyP Region

Two HyP regions corresponding to the acidic and basic subunits were identified for six of the twenty isoforms over all genotypes excluding where Ara h 3.13 was very low in abundance, and thus, HyP peptides are very low and are not detected (refer to Figure 5C, Supplementary Figures S9A-F and supplementary excel). The Ara h 3 acidic subunit HyP region was identified in Ara h 3.4/5/10/13/17/20, which includes all five high-abundance Ara h 3 isoforms and one of the variable isoforms. Interestingly, the most abundant HyP-modified sites align and are present at similar modified to unmodified ratios in each isoform. An additional HyP-modified peptide was identified for Ara h 3.13 adjacent to the primary region, which differs from the other isoforms. However, detection of the corresponding region in the other isoforms is hindered by the additional tryptic site after this proline, releasing a peptide 5 amino acids long, which may be undetected with our methodology. The HyP region in the basic subunit was identified for Ara h 3.10 and Ara h 3.13, but peptides were predominantly identified without HyP. Rabjohn et al. (21) and Rouge et al. (22) mapped linear IgE epitopes of Ara h 3, with each study identifying different epitopes. Neither study identified linear IgE epitopes which cover HyP-containing regions identified here; however, as these studies used overlapping peptides synthesized without HyP modification, they do not address the potential for epitopes in HyP-containing regions. The Rouge et al.'s study (22) did identify epitopes on either side of the Ara h 3 acidic subunit HyP region. Similar studies, using synthetic peptides containing HyP at sites identified in this study, are required to address potential IgE binding. HyP modification is likely influenced by the target protein site localized steric hindrance to prolyl hydroxylase and a recognizable motif sequence. High amounts of HyP vs. unmodified suggest that the conformation of the peanut allergens at these sites is relatively stable and accessible and that these allergens hold a predominant motif for HyP modification.

## Discussion

The peanut genotypes studied were largely similar to one another (Figure 1), but key points of genotype-specific variation may impact various applications. These key points are evident at the level of allergen families, isoforms within each family, as well as HyP modification of allergens. To illustrate, these points will be explored relative to such potential applications.

### Reference Materials and Detection of Food Allergens

With respect to the use of reference materials for allergen detection, genotype variability plays a critical role in ensuring a reference material that is representative of materials used by the food industry. For instance, in the case that an unknown peanut genotype contaminated a food and that contamination was assessed using a reference material of an unknown peanut genotype, the worst-case scenario is that the reference material contains the maximum amount of a specific allergen family compared to a contaminant with the minimum amount of the allergen family. If Ara h 6 was used as the analyte for a detection method, this could result in an approximate 4.3-fold difference in measured and converted total peanut compared to true present peanut. Thus, a sample reporting 1 ppm peanut protein (*via* Ara h 6) relative to the reference material would then be underreporting a 4.3 ppm peanut protein contamination due to the variability of Ara h 6 among peanut genotypes. Similar uncertainty can be applied to Ara h 1, Ara h 2, and Ara h 3 with fold differences up to 2-fold. For detection of peanut from food, Ara h 2 is likely the choice target due to low observed variability but also the propensity of Ara h 1 and Ara h 3 to be affected by food processing, such as roasting (10).

Regarding HyP modification, caution is warranted for detection methods using monoclonal antibodies targeting regions that have multiple sites of HyP modification. Such an antibody would need to be verified to be able to adequately detect the predominant HyP modification sites (Figure 5). As the HyP-rich region of Ara h 2 was not observed without HyP, this would require the antibody to be raised against the HyP-rich epitope. Such considerations also apply to targeted MS-based detection, if the peptide selected is from a known HyP-rich region, then the modification must be present on the stable-isotope labeled peptide (23).

### Food Allergy Diagnosis and Treatment

For an individual being evaluated for peanut allergy or undergoing immunotherapy, it is critical that they are evaluated or treated for the allergenic proteins to which they may be allergic. As the abundance of isoforms Ara h 3.7, Ara h 3.13, and Ara h 3.16 appears to be extremely genotype-dependent compared to that of Ara h 3.4/5/10/17/20, peanut material under- or over-representing these isoforms could hinder diagnosis or treatment of peanut allergy. These isoform level differences may not be apparent depending on the type of characterization method used. Although HyP modification has been implicated in allergic elicitation, the low variability of HyP observed in this study suggests that controlling materials with respect to proline modification is not warranted. However, further research into the role of HyP in allergic reactions is warranted. It should be noted that, for food allergens, the clinical impact of differential HyP modification and the presence of different allergen isoforms is yet to be determined. The importance of measuring potential differences when producing clinical material may change as more information becomes available.

This data presented suggests some core considerations when designing an analytical method for the control of variability in biopharmaceutical preparations of food allergens. These preparations include materials for early introduction, diagnosis, or immunotherapy.

Second, the level of allergenic proteins which may be the most variable should be considered as these may contribute greatly to diagnostic or treatment outcomes, and to safety. For peanut, Ara h 6 and the variable Ara h 3 isoforms best fulfill this criterion. If the source material for a clinical peanut material is controlled and known to be derived from a single genotype, this requirement may be lessened.

Third, considerations should be given to protein modifications known to be of potential or likely relevance to allergy. For peanut, HyP modification is understood to heavily influence IgE binding, at least for Ara h 2 (8). The seeming lack of variability in HyP modification observed may make this analyte less pressing to consider. However, given the differences in HyP modifications of Ara h 2.1 and Ara h 2.2, further investigation is warranted to evaluate immunogenicity such as by skin prick testing.

**Figure 6:**
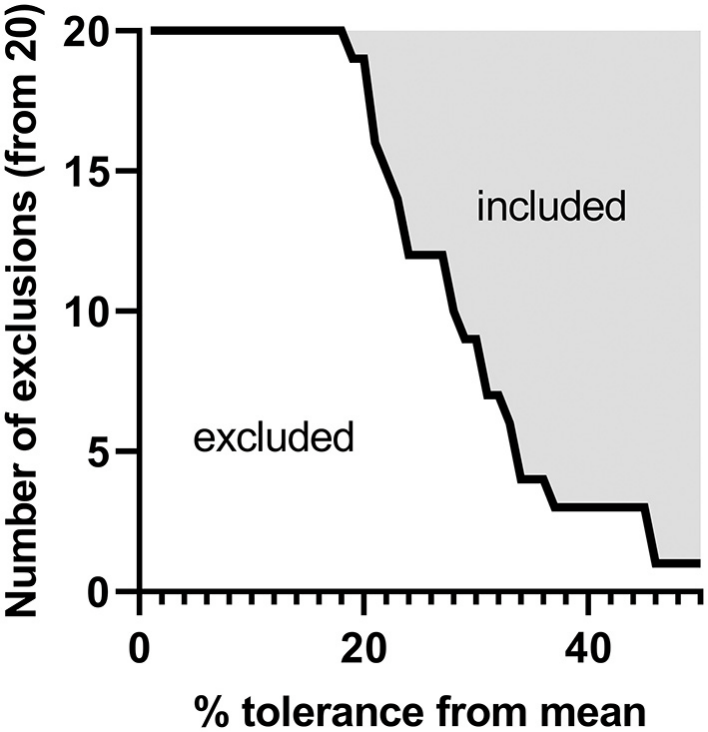
Hypothetical tolerance criteria compared to the variance observed from the 20 peanut genotypes studied. For each of the 20 genotypes studied, the quantities of Ara h 1, 2, 3, and 6 obtained by shared peptide quantitation (percentage peanut protein [allergen (g)/total protein (g)] were compared to the overall mean per allergen family and screened as if they were clinical materials. Genotypes were separated into included or excluded groups per percentage tolerances from 0 to 50% given the relative difference of each of Ara h 1, 2, 3, and 6 to the overall mean per family as well as if any of these allergens were outside of a given tolerance level.

Figure 6 shows an example of an exclusion or inclusion strategy of source material for the clinical use of peanut, using the data in this study (Supplementary Figure S10, for per allergen assessment). In a scenario, where the 20 different peanut genotype samples analyzed represent 20 batches of source material of a peanut biopharmaceutical, the number of excluded samples results from the application of different levels of acceptable variation in each of Ara h 1, 2, 3, and 6 related to the mean of the 20 samples (taken as the reference value). Considering current US and EU regulations of allergen products (24, 25), a 50% variation from the target value may be used by allergen manufacturers, which would include 19 of the 20 tested samples if allergen levels of Ara h 1, 2, 3, and 6 were in the specification. If desired, data from any analyte could be used to set exclusion criteria depending on the intended use of the material and the provenance of the source material.

### Application to Quantitative Risk Assessment

Given that the peanut genotypes studied produce similar amounts of each allergen family, similar isoform ratios, and similar proportions of HyP-modifications, at this moment risk assessment would be unaffected. Peanuts had been variable prior to this study, and quantitative risk assessments related to peanut have erred on the side of overestimating the number of reactions using conservative inputs (26, 27). Thus, the variability and isoform differences observed among peanut genotypes in these data had also been presumably accounted for in risk assessments of peanut. Therefore, nothing needs to change for current food industry risk assessments of peanut.

### Concluding Remarks

The protein composition of different genotypes of peanut was investigated by LC-MS/MS. This approach, combined with the recently published genome of peanut, allowed for the examination of allergen families, isoforms, HyP modifications, and quantities thereof. A comparison of this methodology to previously used RP-HPLC with respect to quantitation of the total amount of Ara h 1, 2, 3, and 6 showed that both techniques were in excellent agreement and that RP-HPLC was suitable for the quantitation of these allergens. However, measurement of variable isoforms of Ara h 3 and of less abundant allergens required the use of LC-MS/MS and could not be achieved using readily available RP-HPLC.

We confidently identified the presence of HyP-modified peptides of Ara h 1 and 3 for the first time and confirmed previous observations of HyP in Ara h 2. The clinical significance of these novel HyP modification sites, and the conclusion that much of the protein in a peanut seed contains HyP, requires further investigation. Proline hydroxylation at the sites observed does not appear to be particularly variable across the genotypes examined. However, some variably expressed isoforms of Ara h 3 likely led to the differences in the amount of HyP-modified Ara h 3 present when using different genotypes.

The use of peanut-containing food products in a variety of applications that require detailed molecular information, ranging from reference materials to clinical diagnostic materials, raises interesting issues with respect to the control of composition for safety and efficacy. The implications are not limited to peanut—the number of allergenic proteins described for major allergenic foods is large and is complicated by the presence of allergen isoforms. We conclude that the overall variability of commercially available peanut seed proteome is low and likely only to impact specific uses. The work presented here describes peptide targets that may be used for more accurate, stable-isotope labeled controlled targeted quantitation of important peanut proteins.

## Data Availability Statement

The datasets presented in this study can be found in online repositories. The names of the repository/repositories and accession number(s) can be found in the article/Supplementary Material.

## Author Contributions

JM: conceptualization, data curation, formal analysis, investigation, methodology, visualization, writing—original draft, and writing—review and editing. LP: data curation, formal analysis, methodology, visualization, and writing—review and editing. SK: resources and writing—review and editing. PJ: conceptualization, formal analysis, funding acquisition, project administration, resources, supervision, visualization, and writing—review and editing. All authors contributed to the article and approved the submitted version.

## Conflict of Interest

The authors declare that the research was conducted in the absence of any commercial or financial relationships that could be construed as a potential conflict of interest.

## Publisher's Note

All claims expressed in this article are solely those of the authors and do not necessarily represent those of their affiliated organizations, or those of the publisher, the editors and the reviewers. Any product that may be evaluated in this article, or claim that may be made by its manufacturer, is not guaranteed or endorsed by the publisher.

## Abbreviations

HyP: hydroxyproline
LC-MS/MS: liquid chromatography- tandem mass spectrometry
RP-HPLC: reverse-phase high-performance liquid chromatography
nsLTP: non-specific lipid-transfer proteins.
