# Supplementary material for: Determination of Allergen Levels, Isoforms, and Their Hydroxyproline Modifications Among Peanut Genotypes by Mass Spectrometry
Source: Front Allergy. 2022 May 24;3:872714. doi: 10.3389/falgy.2022.872714 (PMC9234871; doi:10.3389/falgy.2022.872714)
Supplement: Supplementary file 1 [file Data_Sheet_1.docx]

Contents

[Table S1: Sample description and protein extract concentration 2](#_Toc99539827)

[Table S2: The peptides used in the specific isoform (Unique) peptide quantitation. 3](#_Toc99539828)

[Table S3a-e: Identity matrices of WHO/IUIS peanut allergens to isoforms derived from the peanut genome 5](#_Toc99539829)

[Table S4: Meta-data of the overall number of peptides of the Ara h 3 family and predicted molecular weights 6](#_Toc99539830)

[Table S5a: Peptides used in the closely related isoform (Shared) peptide quantitation of the high-abundance allergens 7](#_Toc99539831)

[Table S5b: Peptides used in the closely related isoform (Shared) peptide quantitation of the mid- and low-abundance allergens 8](#_Toc99539832)

[Table S6: Peptides used in hydroxyproline-peptide assessment. 10](#_Toc99539833)

[Figure S1: Overview of experimental methodology 12](#_Toc99539834)

[Figure S2: Overview of data analysis methodology 13](#_Toc99539835)

[Figure S3: Protein quantitation of Ara h 7, 8, 9, 10 and 11 as determined by closely related isoform (shared) peptide quantitation. 14](#_Toc99539836)

[Figure S4: Comparison of market types within allergen families 15](#_Toc99539837)

[Figure S5: Isoform distribution of Ara h 1, Ara h 2 and Ara h 6, in all genotypes as determined by specific isoform (unique) peptide quantitation. 16](#_Toc99539838)

[Figure S6: Examples of HyP modified and non-modified spectra 17](#_Toc99539839)

[Figure S7a-c: Stacked bar histogram plots show the average peptide quantitation of the hydroxyproline-rich sites in the Ara h 1 isoforms. 20](#_Toc99539840)

[Figure S8a-c: Stacked bar histogram plots show the average peptide quantitation of the hydroxyproline-rich sites in the Ara h 2 isoforms. 21](#_Toc99539841)

[Figure S9a-f: Stacked bar histogram plots show the average peptide quantitation of the hydroxyproline-rich sites in the Ara h 3 isoforms. 23](#_Toc99539842)

[Figure S10: Hypothetical tolerance criteria compared to the variance observed from the 20 peanut genotypes studied per allergen. 24](#_Toc99539843)

# Table S1: Sample description and protein extract concentration

| **Market type** | **Sub- species/Botanical variety^1^** | **Genotype** | **Acronym (in manuscript)** | **Protein Concentration (mg.mL^-1^)** | **Commercial/**  **Experimental variety** |
| --- | --- | --- | --- | --- | --- |
| Runner | *hypogaea/hypogaea* | Exp. 27-1516 | r1 | 21.5 | Experimental |
| Runner | *hypogaea/hypogaea* | Georgia-06G | r2 | 20.7 | Commercial |
| Runner | *hypogaea/hypogaea* | Tifguard | r3 | 19.9 | Commercial |
| Runner | *hypogaea/hypogaea* | Florida-07 | r4 | 21.7 | Commercial |
| Runner | *hypogaea/hypogaea* | Florunner 4/14 | r5 | 17.8 | Commercial |
| Spanish | *fastigiata/vulgaris* | OLin Spanish | s1 | 23.6 | Commercial |
| Valencia | *fastigiata/fastigiata* | Valencia C | v1 | 24.3 | Commercial |
| Valencia | *fastigiata/fastigiata* | Valencia A | v2 | 20.5 | Commercial |
| Valencia | *fastigiata/fastigiata* | H&W 102 | v3 | 17.8 | Commercial |
| Virginia | *hypogaea/hypogaea* | NC9 | vg1 | 14.5 | Commercial |
| Virginia | *hypogaea/hypogaea* | NC 10C | vg2 | 15.6 | Commercial |
| Virginia | *hypogaea/hypogaea* | NC-V 11 | vg3 | 15.9 | Commercial |
| Virginia | *hypogaea/hypogaea* | NC 12C | vg4 | 18.1 | Commercial |
| Virginia | *hypogaea/hypogaea* | Gregory | vg5 | 17.7 | Commercial |
| Virginia | *hypogaea/hypogaea* | Perry | vg6 | 17.7 | Commercial |
| Virginia | *hypogaea/hypogaea* | Phillips | vg7 | 15.8 | Commercial |
| Virginia | *hypogaea/hypogaea* | Brantley | vg8 | 18.3 | Commercial |
| Virginia | *hypogaea/hypogaea* | Bailey | vg9 | 14.9 | Commercial |
| Virginia | *hypogaea/hypogaea* | Sugg | vg10 | 19.8 | Commercial |
| Virginia | *hypogaea/hypogaea* | CHAMPS | vg11 | 19.5 | Commercial |

*1: Based on Bertioli et al, 2011*

Sample description and protein extract concentration as described in Koppelman et al, 2016

# Table S2: The peptides used in the specific isoform (Unique) peptide quantitation.

| **Allergen**  **(No. of isoforms)** | **Name of isoforms in paper**  **Family_Isoform number_Chromosome** | **Protein id or gene position (NCBI)** | **Description in NCBI** | **Tryptic Proteomic**  **Coverage (%)**  **(#Total Peptides )**  **(#Shared Peptides )**  **(#Unique peptides)** | **Peptide sequence** | **m/z** | **z** | **-10LgP** | **RT** |
| --- | --- | --- | --- | --- | --- | --- | --- | --- | --- |
| Ara h 1.1  (1) | Arah1_1_09  (1.01) | NP_001363136.1 | allergen Ara h 1, clone P17 precursor | 61 (42)(31)(11) | DGEPDLSNNFGR | 660.79 | 2 | 78.88 | 40.57 |
|  |  |  |  |  | SSDNEGVIVK | 524.27 | 2 | 76.49 | 32.05 |
|  |  |  |  |  | EGEQEWGTPGSEVR | 780.85 | 2 | 92.36 | 39.09 |
| Ara h 1.2  (1) | Arah1_2_19  (1.02) | NP_001363139.1 | allergen Ara h 1, clone P41B precursor | 60 (44)(31)(13) | IVQIEAKPNTLVLPK | 832.01 | 2 | 68.88 | 46.14 |
|  |  |  |  |  | EGEQEWGTPGSHVR | 784.86 | 2 | 113.65 | 37.02 |
|  |  |  |  |  | SSENNEGVIVK | 588.30 | 2 | 78.39 | 31.75 |
| Ara h 2.1  (1) | Arah2_1_08  (2.01) | NP_001363146.1 | conglutin-7-like precursor | 74 (22)(15)(7) | C(+57.02)DLDVESGGR | 554.24 | 2 | 65.87 | 32.37 |
|  |  |  |  |  | - |  |  |  |  |
|  |  |  |  |  | - |  |  |  |  |
| Ara h 2.2  (1) | Arah2_2_18  (2.02) | XP_025671514.1 | conglutin-7-like | 75 (19)(15)(4) | C(+57.02)DLEVESGGR | 561.25 | 2 | 64.24 | 32.51 |
|  |  |  |  |  | - |  |  |  |  |
|  |  |  |  |  | - |  |  |  |  |
| Ara h 3.1  (1) | Arah3_1_02  (3.01) | XP_025644723.1 | legumin type B isoform X1 | 67 (29)(25)(4) | ISSANSLTFPILR | 709.90 | 2 | 67.54 | 53.26 |
|  |  |  |  |  | HGQHQQDEYGSQGEEEGNNVLSGFSTQLLAHAFGVDEEIAR | 1121.77 | 4 | 171.92 | 61.44 |
|  |  |  |  |  | - |  |  |  |  |
| Ara h 3.2/12  (2) | Arah3_2_02,  Arah3_12_12  (3.02/12) | XP_025650075.1  XP_025633145.1 | legumin B  legumin B | 57 (16)(12)(4)  45 (17)(12)(5) | AGSDAFDWVAIK | 640.32 | 2 | 75.63 | 52.97 |
|  |  |  |  |  | GVMEIVVTGC(+57.02)R | 610.81 | 2 | 76.95 | 45.79 |
|  |  |  |  |  | TSDNPIINTLAGELSLVR^1^ | 957.02 | 2 | 99.51 | 65.49 |
| Ara h 3.3/6  (2) | Arah3_3_06^1^,  Arah3_6_06^2^  (3.03/06) | XP_025603245.1  XP_025607222.1 | LOC112695204  arachin Ahy-3-like | 69 (21)(17)(4)  69 (21)(17)(4) | GLSILVPAER | 527.82 | 2 | 49.13 | 49.44 |
|  |  |  |  |  | - |  |  |  |  |
|  |  |  |  |  | - |  |  |  |  |
| Ara h 3.4/5  (2) | Arah3_4_06^3^,  Arah3_5_06^4^  (3.04/05) | XP_025603242.1  XP_025603240.1 | arachin Ahy-3-like  arachin Ahy-3-like | 75 (34)(24)(10)  75 (34)(24)(10) | SQSDNFEYVAFK | 717.83 | 2 | 86.73 | 46.09 |
|  |  |  |  |  | GENESEEEGAIVTVK | 795.88 | 2 | 101.60 | 39.55 |
|  |  |  |  |  | FFVPPSQQSLR | 653.35 | 2 | 87.72 | 45.75 |
| Ara h 3.7  (1) | Arah3_7_06  (3.07) | XP_025603243.1 | arachin Ahy-3-like | 72 (28)(18)(10) | ILNPDEEDESSR | 702.32 | 2 | 84.65 | 33.94 |
|  |  |  |  |  | QGGEENEC(+57.02)QFQR | 741.31 | 2 | 64.81 | 29.77 |
|  |  |  |  |  | GEEQENEGNNIFSGFAQEFLQHAFQVDR | 810.87 | 4 | 200 | 68.56 |
| Ara h 3.8/15  (2) | Arah3_8_06  Arah3_15_16  (3.08/15) | XP_025603244.1  XP_025663188.1 | 11S globulin  11S globulin | 45 (16)(14)(2)  42 (15)(14)(1) | LPILADLQLSAER | 719.92 | 2 | 64.01 | 55.44 |
|  |  |  |  |  | NIVMVEGGLDVVRPEPGSR | 675.36 | 3 | 92.96 | 47.60 |
|  |  |  |  |  | FYIAGNTEDEHGEGGR | 584.59 | 3 | 85.14 | 38.93 |
| Ara h 3.9  (1) | Arah3_9_06^5^  (3.09) | XP_025603268.1 | LOC112695229 | 64 (19)(15)(4) | TVAESLGIDMGIAGK | 731.38 | 2 | 77.00 | 49.32 |
|  |  |  |  |  | LTAEEAINLK | 551.31 | 2 | 56.81 | 44.94 |
|  |  |  |  |  | LVALEPSK | 428.76 | 2 | 43.81 | 38.86 |
| Ara h 3.10  (1) | Arah3_10_06  (3.10) | XP_025603315.1 | arachin Ahy-3-like | 81 (39)(27)(12) | TDSRPSIANLAGENSVIDNLPEEVVANSYGLPR | 875.19 | 4 | 200.00 | 55.85 |
|  |  |  |  |  | GENESEEEGAIVTVR | 809.88 | 2 | 87.56 | 39.85 |
|  |  |  |  |  | FFVPPSQQSPR | 645.34 | 2 | 83.41 | 40.70 |
| Ara h 3.11  (1) | Arah3_11_12  (3.11) | XP_025632438.1 | legumin type B | 57 (27)(25)(2) | ISSVNSLTFPILR | 723.92 | 2 | 52.28 | 55.92 |
|  |  |  |  |  | - |  |  |  |  |
|  |  |  |  |  | - |  |  |  |  |
| Ara h 3.13  (1) | Arah3_13_16  (3.13) | XP_025663039.1 | arachin Ahy-3-like | 79 (37)(23)(14) | AGQEQENEGGNIFSGFTSEFLAQAFQVDDR | 1097.83 | 3 | 125.94 | 67.56 |
|  |  |  |  |  | FFVPPFQQSPR | 450.57 | 3 | 87.94 | 49.67 |
|  |  |  |  |  | SPDEEEEYDEDEYAEEER | 1131.92 | 2 | 97.33 | 38.31 |
| Ara h 3.14  (1) | Arah3_14_16^5^  (3.14) | XP_025663119.1 | LOC112758621 | 64 (19)(15)(4) | TVAESLDIDMGIAGK | 760.39 | 2 | 97.15 | 49.21 |
|  |  |  |  |  | LTAEEAISLK | 537.81 | 2 | 50.64 | 43.25 |
|  |  |  |  |  | LVALEPTK | 435.77 | 2 | 49.27 | 37.93 |
| Ara h 3.16  (1) | Arah3_16_16^6^  (3.16) | XR_003179792.1 | LOC112758683 | 79 (33)(21)(12) | FYLAGNQEQEFLR | 807.90 | 2 | 89.89 | 50.00 |
|  |  |  |  |  | ILSPDEEDESSR | 688.81 | 2 | 93.62 | 33.09 |
|  |  |  |  |  | FQVGQDDPSQQQQDSHQK | 1050.47 | 2 | 102.00 | 33.79 |
| Ara h 3.17/20  (2) | Arah3_17_16^7^,  Arah3_20_16^8^  (3.17/20) | XP_025663190.1  XP_025663194.1 | arachin Ahy-3-like  arachin Ahy-3-like | 76 (37)(19)(18)  76 (37)(19)(18) | VFDEELQEGHVLVVPQNFAVAGK | 1263.16 | 2 | 200.00 | 52.18 |
|  |  |  |  |  | QILQNLR | 442.77 | 2 | 38.33 | 41.53 |
|  |  |  |  |  | GENESDEQGAIVTVR | 802.38 | 2 | 103.29 | 38.05 |
| Ara h 3.18  (1) | Arah3_18_16  (3.18) | XP_025663195.1 | arachin Ahy-3-like | 64 (21)(3)(18) | EIVQNLR | 436.25 | 2 | 41.78 | 34.60 |
|  |  |  |  |  | IDSEGGFIETWNPK | 796.88 | 2 | 82.65 | 47.76 |
|  |  |  |  |  | QEQEFLQYQHQHGGPR | 496.24 | 4 | 87.18 | 36.16 |
| Ara h 3.19  (1) | Arah3_19_16  (3.19) | NP_001363142.1 | arachin Ahy-3 precursor | 55 (19(15)(4) | GGLSILVPPEWR | 662.37 | 2 | 62.00 | 56.04 |
|  |  |  |  |  | HFQGQDQSQRPLDTHQK | 513.25 | 4 | 64.79 | 29.56 |
|  |  |  |  |  | - |  |  |  |  |
| Ara h 6.1  (1) | Arah6_1_08  (6.01) | XP_025614701.1 | conglutin-like | 69 (17)(15)(2) | C(+57.02)C(+57.02)DELDQMENTER | 850.32 | 2 | 76.67 | 38.94 |
|  |  |  |  |  | - |  |  |  |  |
|  |  |  |  |  | - |  |  |  |  |
| Ara h 6.2  (1) | Arah6_2_18  (6.02) | NP_001363141.1 | conglutin precursor | 69 (17)(15)(2) | C(+57.02)C(+57.02)DELNEMENTQR | 849.82 | 2 | 118.81 | 39.29 |
|  |  |  |  |  | - |  |  |  |  |
|  |  |  |  |  | - |  |  |  |  |
| Ara h 7.1  (1) | Arah7_1_08  (7.01) | XR_003156017.2 | conglutin-like | 58 (11)(9)(2) | VEQEQEQEQDEYPYIQR | 1092.97 | 2 | 82.56 | 35.92 |
|  |  |  |  |  |  |  |  |  |  |
|  |  |  |  |  |  |  |  |  |  |
| Ara h 7.2  (1) | Arah7_2_18  (7.02) | XR_003187481.2 | conglutin-like | 57 (10)(9)(1) | VEQEQEQEQDEYPYSQR | 1092.97 | 2 | 82.56 | 35.92 |
|  |  |  |  |  |  |  |  |  |  |
|  |  |  |  |  |  |  |  |  |  |
| GlyP  (1) | - | P00489.3 | Rabbit glycogen phosphorylase, muscle form | 63 (45)(45)(45) | TC(+57.02)AYTNHTVLPEALER | 625.64 | 3 | 86.12 | 44.11 |
|  |  |  |  |  | VIFLENYR | 527.29 | 2 | 44.20 | 46.89 |
|  |  |  |  |  | VLVDLER | 422.25 | 2 | 25.90 | 41.99 |

Table describes the protein/allergen origin and characteristics of the specific isoform (unique) peptide used in quantitation. Protein origin (NCBI ID or gene position) are listed with tryptic coverage (%) giving Total Peptides, Shared Peptides and Unique peptides. Tryptic peptide sequence used (with carbamidomethylation modification indicated: +57.02), m/z and z of peptide and retention time (RT) is also shown. Allergen isoforms that were unable to be distinguished are colored red. ^1^Mature protein identical to 3.6. ^2^Mature protein identical to 3.3. ^3^Mature protein identical to 3.5. ^4^Mature protein identical to 3.4. ^5^Incorrect genome splicing annotation (No LC-MS/MS proof of our sequence or NCBIs). ^6^Incorrect genome start annotation, identified as a pseudogene. (LC-MS/MS proof of our sequence). ^7^Mature protein identical to 3.20. ^8^Mature protein identical to 3.17.

# Table S3a-e: Identity matrices of WHO/IUIS peanut allergens to isoforms derived from the peanut genome

**Table S3a: Low identity (<50%) Ara h 3 Identity matrix**

| **IUIS Allergen**  **Nomenclature** | **Allergen Nomenclature (This Paper)** | | | | | | | |
| --- | --- | --- | --- | --- | --- | --- | --- | --- |
|  | **3.9** | **3.14** | **3.2** | **3.12** | **3.8** | **3.15** | **3.1** | **3.11** |
| **3_0201** | 37.8 | 38.0 | 35.3 | 35.4 | 41.0 | 40.9 | 42.3 | 41.7 |
| **3_0101** | 38.6 | 38.6 | 36.8 | 36.4 | 42.8 | 42.7 | 44.9 | 44.9 |

**Table S3b: High identity (>50%) Ara h 3 Identity matrix**

| **IUIS Allergen Nomenclature** | **Allergen Nomenclature (This Paper)** | | | | | | | | | | | |
| --- | --- | --- | --- | --- | --- | --- | --- | --- | --- | --- | --- | --- |
|  | **3.18** | **3.19** | **3.3** | **3.6** | **3.13** | **3.17** | **3.20** | **3.4** | **3.5** | **3.10** | **3.7** | **3.16** |
| **3_0201** | 71.8 | 70.6 | 73.2 | 70.7 | 92.8 | 90.0 | 89.9 | 91.7 | 91.7 | 93.8 | 74.7 | 71.3 |
| **3_0101** | 72.3 | 71.1 | 73.83 | 72.0 | 93.4 | 93.5 | 93.5 | 94.8 | 94.8 | 97.6 | 74.4 | 73.6 |

**Table S3c: Ara h 2 Identity matrix**

| **IUIS Allergen Nomenclature** | **Allergen Nomenclature (This Paper)** | |
| --- | --- | --- |
|  | **2.1** | **2.2** |
| **2_0101** | 100.0 | 98.7 |
| **2_0201** | 98.8 | 100.0 |

**Table S3d: Ara h 1 Identity matrix**

| **IUIS Allergen Nomenclature** | **Allergen Nomenclature (This Paper)** | |
| --- | --- | --- |
|  | **1.2** | **1.1** |
| **1_0101** | 100.0 | 96.9 |

**Table S3e: Ara h 6 Identity matrix**

| **IUIS Allergen Nomenclature** | **Allergen Nomenclature (This Paper)** | |
| --- | --- | --- |
|  | **6.1** | **6.2** |
| **6_0101** | 92.3 | 94.6 |

Tables S3a and b shows the identity of Ara h 3 isoforms. Table S3c shows the identity of Ara h 2 isoforms. Table S3d shows the identity of Ara h 1 isoforms. Table S3e shows the identity of Ara h 6 isoforms.

Blue Highlighted cell indicates highest identity of genomic derived isoforms to WHO/IUIS allergen isoform

# Table S4: Meta-data of the overall number of peptides of the Ara h 3 family and predicted molecular weights

| **Shared peptide quantitation** | | |
| --- | --- | --- |
| **Protein** | **Number of peptides found experimentally** | **Closely Related/Specific** |
| Ara h 3.4/5/10/13/17/20 | 17 | Closely Related |
| Ara h 3.1/11 | 25 | Closely Related |
| Ara h 3.8/15 | 14 | Closely Related |
| Ara h 3.9/14 | 15 | Closely Related |
| Ara h 3.2/12 | 12 | Closely Related |
| Ara h 3.3/6/19 | 10 | Closely Related |
| Ara h 3.7/16 | 15 | Closely Related |
| Ara h 3.18 | 18 | Specific |

| **Unique peptide quantitation** | | | | | |
| --- | --- | --- | --- | --- | --- |
| **Protein** | **Protein monoisotopic weight** | **Acidic subunit monoisotopic weight** | **Number of peptides found experimentally** | **Closely Related/Specific** | **Notes** |
| Ara h 3.4/5 | 60197/60197 | 39667/39667 | 10 | Closely Related | No unique peptides |
| Ara h 3.10 | 60595 | 40081 | 12 | Specific |  |
| Ara h 3.13 | 60339 | 39689 | 14 | Specific |  |
| Ara h 3.17/20 | 61459/61459 | 40833/40833 | 18 | Closely Related | No unique peptides |
| Ara h 3.1 | 72916 | 52416 | 4 | Specific |  |
| Ara h 3.11 | 71588 | 50970 | 2 | Specific |  |
| Ara h 3.8/15 | 50732/50453 | 29876/29726 | 14 | Closely Related | Specific peptides of 8 and 15 exist, but low abundant |
| Ara h 3.9 | 47816 | 27529 | 4 | Specific |  |
| Ara h 3.14 | 47816 | 27579 | 4 | Specific |  |
| Ara h 3.2/12 | 51416/51596 | 31084/31294 | 12 | Closely Related | Specific peptides of 2 and 12 exist, but low abundant |
| Ara h 3.3/6 | 51144/45846 | 30640/25342 | 4 | Closely Related | No unique peptides.  Ara h 3.6 is a probable pseudogene |
| Ara h 3.19 | 54535 | 33990 | 4 | Specific |  |
| Ara h 3.7 | 58364 | 37703 | 10 | Specific |  |
| Ara h 3.16 | 58312 | 37651 | 12 | Specific |  |
| Ara h 3.18 | 52311 | 31236 | 18 | Specific |  |

Table S4 gives an overview of the number of Ara h 3 peptides that could be used for shared and unique peptide quantification. Allergen isoforms that were unable to be distinguished are colored red. Monoisotopic weight was determined using the compute Mw tool (Expasy) (Gasteiger et al, 2005) of the sequences previously published in Marsh et al (2020). The acidic and basic subunit cleavage site was determined as occurring after the Asn amino-acid in the consensuses sequence N**↓**G(V/I/L)(E/Q/D)ET(V/I/L)C.

# Table S5a: Peptides used in the closely related isoform (Shared) peptide quantitation of the high-abundance allergens

| **Allergen**  **(No. of isoforms)** | **Name of isoforms** | **Protein id or gene position (NCBI)** | **Peptide sequence** | **m/z** | **z** | **-10lgP** | **RT** |
| --- | --- | --- | --- | --- | --- | --- | --- |
| Ara h 1 (2) | Arah1_1_09:Arah1_2_19  (1.1/2) | NP_001363136.1  NP_001363139.1 | GTGNLELVAVR | 564.82 | 2 | 72.13 | 45.55 |
|  |  |  | NTLEAAFNAEFNEIR | 869.92 | 2 | 103.84 | 54.94 |
|  |  |  | SFNLDEGHALR | 629.81 | 2 | 86.25 | 43.25 |
| Ara h 2 (2) | Arah2_1_08:Arah2_2_18  (2.1/2) | NP_001363146.1  XP_025671514.1 | ANLRPC(+57.02)EQHLMQK | 542.27 | 3 | 99.33 | 36.50 |
|  |  |  | C(+57.02)C(+57.02)NELNEFENNQR | 863.85 | 2 | 76.96 | 40.80 |
|  |  |  | NLPQQC(+57.02)GLR | 543.28 | 2 | 58.52 | 35.90 |
| Ara h 3 (20) | Arah3_1_02:Arah3_11_12  (3.1/11) | XP_025644723.1  XP_025632438.1 | EGQILLVPQNFAVGK | 806.96 | 2 | 78.35 | 52.01 |
|  |  |  | FYLAGNPEEEHPETQQQQPQTR | 876.41 | 3 | 110.58 | 42.11 |
|  |  |  | IESQGGITETWNSNHPELR | 723.35 | 3 | 94.89 | 42.72 |
|  | Arah3_2_02:Arah3_12_12  (3.2/12) | XP_025650075.1  XP_025633145.1 | AGSDAFDWVAIK | 640.32 | 2 | 75.63 | 52.97 |
|  |  |  | GVMEIVVTGC(+57.02)R | 610.81 | 2 | 76.95 | 45.79 |
|  |  |  | TSDNPIINTLAGELSLVR | 957.02 | 2 | 99.51 | 65.49 |
|  | Arah3_3_06:Arah3_6_06:Arah3_19_16  (3.3/6/19) | XP_025603245.1  XP_025607222.1  NP_001363142.1 | LGLSAEYGSIHR | 651.84 | 2 | 74.60 | 43.05 |
|  |  |  | SQSEHFLYVAFK | 728.37 | 2 | 88.13 | 47.64 |
|  |  |  | TVNELDLPILNR | 698.89 | 2 | 69.02 | 52.91 |
|  | Arah3_4_06:Arah3_5_06:Arah3_10_06:Arah3_13_16:Arah3_17_16:Arah3_20_16  (3.4/5/10/13/17/20) | XP_025603242.1  XP_025603240.1  XP_025603315.1  XP_025663039.1  XP_025663190.1  XP_025663194.1 | FNLAGNHEQEFLR | 787.89 | 2 | 99.77 | 45.49 |
|  |  |  | RPFYSNAPQEIFIQQGR | 513.52 | 4 | 112.44 | 47.40 |
|  |  |  | SPDIYNPQAGSLK | 695.35 | 2 | 87.49 | 40.91 |
|  | Arah3_7_06:Arah3_16_16  (3.7/16) | XP_025603243.1  XR_003179792.1 | AQSENYEYLAFK | 731.85 | 2 | 83.24 | 46.54 |
|  |  |  | SSNPDIYNPQAGSLR | 809.90 | 2 | 92.25 | 41.90 |
|  |  |  | VYDEELQEGHVLVVPQNFAVAAK | 1278.16 | 2 | 137.88 | 51.56 |
|  | Arah3_8_06:Arah3_15_16  (3.8/15) | XP_025603244.1  XP_025663188.1 | LPILADLQLSAER | 719.92 | 2 | 64.01 | 55.44 |
|  |  |  | NIVMVEGGLDVVRPEPGSR | 675.36 | 3 | 92.96 | 47.60 |
|  |  |  | FYIAGNTEDEHGEGGR | 584.59 | 3 | 85.14 | 38.93 |
|  | Arah3_9_06:Arah3_14_16  (3.9/14) | XP_025603268.1  XP_025663119.1 | EGQILIVPQQFVVAK | 834.99 | 2 | 76.48 | 54.11 |
|  |  |  | GLLLPHYINAPR | 455.27 | 3 | 58.47 | 48.72 |
|  |  |  | NDQFQC(+57.02)VGVSALR | 747.36 | 2 | 94.10 | 44.35 |
|  | Arah3_18_16  (3.18/-) | XP_025663195.1 | EIVQNLR | 436.25 | 2 | 41.78 | 34.60 |
|  |  |  | IDSEGGFIETWNPK | 796.88 | 2 | 82.65 | 47.76 |
|  |  |  | QEQEFLQYQHQHGGPR | 496.24 | 4 | 87.18 | 36.16 |
| Ara h 6 (2) | Arah6_1_08:Arah6_2_18  (6.1/2) | XP_025614701.1  NP_001363141.1 | C(+57.02)DLDVSGGR | 489.72 | 2 | 70.51 | 30.58 |
|  |  |  | ELMNLPQQC(+57.02)NFR | 775.37 | 2 | 80.30 | 45.57 |
|  |  |  | VNLKPC(+57.02)EQHIMQR | 551.62 | 3 | 86.06 | 38.79 |

# Table S5b: Peptides used in the closely related isoform (Shared) peptide quantitation of the mid- and low-abundance allergens

| **Allergen**  **(No. of isoforms)** | **Name of isoforms**  **(bold indicates Uniprot accession highest identity)** | **Protein id or gene position (NCBI)** | **Peptide sequence** | **m/z** | **z** | **-10LgP** | **RT** |
| --- | --- | --- | --- | --- | --- | --- | --- |
| Ara h 5 (5) | Arah5_1_13  Arah5_2_13  Arah5_3_05  Arah5_4_05  Arah5_5_15  Arah5_6_15  Arah5_7_03  Arah5_8_03 | XP_025644027.1  XP_025644029.1  XP_025700849.1  NP_001363134.1  XP_025656159.1  XP_025656160.1  XP_025688222.1  XP_025688220.1 | ND | | | | |
| Ara h 7 (2) | Arah7_1_08:Arah7_2_18  (7.1/2) | XR_003156017.2  XR_003187481.2 | ANLRPC(+57.02)EEHIR | 465.57 | 3 | 83.56 | 30.96 |
|  |  |  | C(+57.02)MC(+57.02)QALQQILQNQSFR | 1012.98 | 2 | 107.61 | 55.68 |
|  |  |  | NLPQNC(+57.02)GFR | 553.26 | 2 | 69.00 | 36.57 |
| Ara h 8 (2) | Arah8_1_14:Arah8_9_04  (8.1/9) | XP_025646545.1  XP_025694603.1 | GDAPLPDEVHQDVK | 507.25 | 3 | 76.37 | 39.18 |
|  |  |  | GVHTFEEESTSPVPPAK | 604.63 | 3 | 83.08 | 39.43 |
|  |  |  |  |  |  |  |  |
| Ara h 8 (28) | Arah8_18_11:Arah8_19_11:Arah8_20_11:Arah8_21_11:Arah8_22_11:Arah8_23_11:Arah8_24_11:Arah8_25_11:Arah8_26_11:Arah8_28_11:Arah8_29_11:Arah8_30_11:Arah8_31_11:Arah8_32_11:Arah8_33_11:Arah8_34_11:Arah8_35_01:Arah8_36_01:Arah8_37_01:Arah8_38_01:Arah8_39_01:Arah8_40_01:Arah8_41_01:Arah8_42_01:Arah8_43_01:Arah8_44_01:Arah8_45_17:Arah8_46_07  (8.18/19/20/21/22/23/24/25/26/28/29/30/31/32/33/34/35/36/36/37/38/39/40/41/42/43/44/45/46) | XP_025630152.1  XP_025630154.1  XP_025630155.1  XP_025630259.1  XP_025630260.1  XP_025630262.1  XP_025630266.1  XP_025630265.1  XP_025630264.1  XP_025630267.1  XP_025630269.1  XP_025630270.1  XP_025630272.1  XP_025630286.1  XP_025630287.1  XP_025609884.1  XP_025609905.1  XP_025609896.1  XP_025609919.1  XP_025609931.1  XP_025609943.1  XP_025610803.1  XP_025610823.1  XP_025610813.1  XP_025610791.1  XP_025665897.1  LOC112702190 | SVEIVEGNGGPGTIK | 728.89 | 2 | 80.17 | 40.53 |
|  |  |  |  |  |  |  |  |
|  |  |  |  |  |  |  |  |
| Ara h 8 (13) | Arah8_2_14  Arah8_3_14  Arah8_4_14  Arah8_6_14  Arah8_7_14  Arah8_8_14  Arah8_10_04  Arah8_11_04  Arah8_12_04  Arah8_14_04  Arah8_15_04  Arah8_16_04  Arah8_17_11  Arah8_27_11 | XP_025646542.1  XP_025646543.1  XP_025646544.1  XP_025646549.1  XP_025646548.1  XP_025644849.1  XP_025694590.1  XP_025694601.1  XP_025694602.1  XR_003199147.1  XP_025694606.1  XP_025692115.1  QHO19281.1  XP_025630268.1 | ND | | | | |
| Ara h 9 (1) | Arah9_4_02  (9.4/-) | XP_025656642.1 | GGVPSGPC(+57.02)C(+57.02)SGVR | 645.29 | 2 | 49.88 | 33.18 |
|  |  |  | AAAGSLHGLNQGNAAALPGR | 923.49 | 2 | 84.70 | 39.96 |
|  |  |  |  |  |  |  |  |
| Ara h 9 (2) | Arah9_6_02:Arah9_13_12  (9.6/13) | XP_025626635.1  XP_025633953.1 | SIAPC(+57.02)FGYLK | 578.30 | 2 | 54.01 | 49.09 |
|  |  |  |  |  |  |  |  |
|  |  |  |  |  |  |  |  |
| Ara h 9 (2) | Arah9_19_14:Arah9_20_04  (9.19/20) | QHO08204.1  XP_025695791.1 | TLLSLAPAR | 471.29 | 2 | 38.23 | 46.10 |
|  |  |  | GINFNQNTLNLAAGLPSK | 936.50 | 2 | 89.02 | 51.80 |
|  |  |  |  |  |  |  |  |
| Ara h 9 (20) | Arah9.1_02  Arah9.2_02  Arah9.3_02  Arah9.5_02  Arah9.7_02  Arah9.8_02  Arah9.9_12  Arah9.10_12  Arah9.11_12  Arah9.12_12  Arah9.15_12  Arah9.16_12  Arah9.17_12  Arah9.18_14  Arah9.21_04  Arah9.22_06  Arah9.23_16  Arah9.24_07  Arah9.25_18 | XP_025656457.1  XP_025656469.1  XP_025656480.1  XP_025626657.1  XP_025626648.1  XP_025626667.1  XP_025633971.1  XP_025633929.1  XP_025633930.1  XP_025633952.1  LOC112729389  XP_025635400.1  XP_025635401.1  XP_025645001.1  XP_025695790.1  XP_025606139.1  XP_025658854.1  XP_025610112.1  XP_025673800.1 | ND | | | | |
| Ara h 10 (2) | Arah10_1_05: Arah10_2_05  (10.1/2) | XP_025700683.1  XP_025656332.1 | APHQVQVHTPTTQR | 400.71 | 4 | 61.38 | 29.64 |
|  |  |  |  |  |  |  |  |
|  |  |  |  |  |  |  |  |
| Ara h 10 (2) | Arah10_3_06:Arah10_4_16  (10.3/4) | XP_025606204.1  XP_025658931.1 | MADVAGYVGQK | 569.78 | 2 | 76.22 | 39.36 |
|  |  |  | QTHGSVPEQLEMAK | 518.93 | 3 | 75.96 | 38.92 |
|  |  |  |  |  |  |  |  |
| Ara h 11 (2) | Arah11_1_13:Arah11_2_01 | XP_025644435.1  XP_025616575.1 | ND | | | | |
| Ara h 11 (2) | Arah11_3_08:Arah11_4_17  (11.3/4) | XP_025613360.1  XP_025664754.1 | TGYGGGGSYGSSYGGGGTYGSSYGTSYDPSTNQPIR | 1172.50 | 3 | 91.70 | 42.81 |
|  |  |  |  |  |  |  |  |
|  |  |  |  |  |  |  |  |
| Ara h 11 (2) | Arah11_5_10: Arah11_6_20  (11.5/6) | XP_025624254.1  XP_025683566.1 | HPPGANQLDTAR | 426.22 | 3 | 66.35 | 28.53 |
|  |  |  |  |  |  |  |  |
|  |  |  |  |  |  |  |  |

Table S5 describes the protein/allergen origin and characteristics of the shared peptide used in quantitation. Protein origin (NCBI ID or gene position) are listed. Allergen family, number of isoforms in family detected by these peptides are also listed. Tryptic peptide sequence used (with carbamidomethylation modification indicated: +57.02), m/z and z of peptide and retention time (RT) is also shown. . -10LogP probability scores reported by PEAKS v8.5 indicates the scoring significance of a peptide-spectrum match ND indicates no peptide detected for these allergenic isoforms.

# Table S6: Peptides used in hydroxyproline-peptide assessment.

| **Name of isoforms** | **Proline-Hyp Amino acid numbers** | **Acronym** | **Peptide sequence** | **Mean Abundance (fmol)^1^**  **(St.Dev)^2^ (CV)^3^** | **Mean Percent Detected^4^**  **(St.Dev)^5^(CV)^6^** | **m/z** | **z** | **-10LgP** | **RT (min)** | **No. of Spectra** | **Ascore** |
| --- | --- | --- | --- | --- | --- | --- | --- | --- | --- | --- | --- |
| **1.1** | 111 | 1.1-a-HyP 111 | WGP(+15.99)AEPR | 11.8  (2.8)(23.4) | 4.8  (1.2)(25.1) | **414.703** | **2** | **24.8** | **28.4** | **31** | P3:HyP:32.69 |
|  | - | 1.1-a | **WGPAEPR** | 241.8  (64.4)(26.6) | 95.2  (1.2)(1.3) | **406.706** | **2** | **37.9** | **34.9** | **73** | - |
|  | 155 | 1.1-b-HyP 155 | EGEQEWGTP(+15.99)GSEVR | 32.3  (8.5)(26.3) | 12.8  (2.6)(20.5) | **788.846** | **2** | **62.0** | **35.6** | **148** | P9:HyP:1000.00 |
|  | - | 1.1-b | **EGEQEWGTPGSEVR** | 223.8  (52.9)(23.6) | 87.2  (2.6)(3.0) | **780.849** | **2** | **73.8** | **40.1** | **153** | - |
| **1.2** | 594/597 | 1.2-a-HyP 594/597 | **ESHFVSARPQSQSQSP(+15.99)SSP(+15.99)EK** | 21.3  (6.5)(30.7) | 100  (0.0)(0.0) | **583.775** | **4** | **82.44** | **27.02** | **141** | P16:HyP:71.72;  P19:HyP:94.89 |
|  | **-** | 1.2-a | ESHFVSARPQSQSQSPSSPEK | 0 | - | **-** | **-** | **-** | **-** | **-** | - |
| **2.1** | 67/74/76 | 2.1-a-HyP 67/74/76 | DPYSP(+15.99)SQDPYSP(+15.99)SP(+15.99)YDR | 5.3  (1.5)(28.3) | 9.7  (2.2)(23.0) | **1009.914** | **2** | **52.27** | **35.76** | **32** | P5:HyP:87.62;  P12:HyP:56.34;  P14:HyP:61.30 |
|  | 67/74 | 2.1-a-HyP 67/74 | **DPYSP(+15.99)SQDPYSP(+15.99)SPYDR** | 48.3  (11.6)(24.1) | 86.9  (1.9)(2.2) | **1001.918** | **2** | **74.70** | **37.66** | **156** | P5:HyP:101.94;  P12:HyP:55.92 |
|  | 74 | 2.1-a-HyP 74 | DPYSPSQDPYSP(+15.99)SPYDR | 2.3  (1.6)(69.4) | 3.3  (2.6)(77.9) | **993.9183** | **2** | **45.80** | **39.28** | **47** | P12:HyP:11.10 |
|  | - | 2.1-a | DPYSPSQDPYSPSPYDR | 0 | - | **-** | **-** | **-** | **-** | **-** | - |
| **2.2** | 67/74 | 2.2-a-HyP 67/74 | **DPYSP(+15.99)SQDPYSP(+15.99)SQDPDR** | 4.5  (1.3)(28.6) | 100  (0.0)(0.0) | **694.9557** | **3** | **50.54** | **35.58** | **113** | P5:HyP:22.45;  P12:HyP:47.09 |
|  | - | 2.2-a | DPYSPSQDPYSPSQDPDR | 0 | - | **-** | **-** | **-** | **-** | **-** | - |
|  | 86/88 | 2.2-b-HyP 86/88 | DPYSP(+15.99)SP(+15.99)YDR | 27.0  (6.1)(22.5) | 26.8  (6.3)(23.5) | **614.7571** | **2** | **32.91** | **29.72** | **7** | P5:HyP:92.25;  P7:HyP:96.93 |
|  | 86 | 2.2-b-HyP 86 | **DPYSP(+15.99)SPYDR** | 76.3  (20.3)(26.7) | 72.4  (6.1)(8.4) | **606.7619** | **2** | **40.59** | **32.37** | **124** | P5:HyP:60.92 |
|  | - | 2.2-b | DPYSPSPYDR | 0.9  (0.6)(66.4) | 0.8  (0.5)(56.1) | **598.7637** | **2** | **25.99** | **36.73** | **21** | - |
| **3.4/5/10** | 223/226 | 3.4/5/10-a-HyP 223/226 | **SLPYSP(+15.99)YSP(+15.99)QSQPR** | 632.5  (119.1)(19.1) | 75.4  (9.2)(12.2) | **546.9288** | **3** | **58.60** | **37.40** | **116** | P6:HyP:44.84;  P9:HyP:38.03 |
|  | 226 | 3.4/5/10-a-HyP 226 | SLPYSPYSP(+15.99)QSQPR | 190.5  (85.9)(45.1) | 21.9  (7.8)(35.5) | **541.5984** | **3** | **62.96** | **39.98** | **142** | P9:HyP:31.27 |
|  | - | 3.4/5/10-a | SLPYSPYSPQSQPR | 23.7  (17.8)(75.0) | 2.7  (1.8)(64.7) | **803.8954** | **2** | **66.22** | **42.55** | **130** | - |
| **3.10** | 526 | 3.10-b-HyP 526 | FFVPPSQQSP(+15.99)R | 45.2  (10.9)(24.0) | 21.4  (4.0)(18.5) | **653.3326** | **2** | **64.70** | **42.04** | **80** | P10:HyP:56.99 |
|  | - | 3.10-b | **FFVPPSQQSPR** | 167.6  (41.6)(24.8) | 78.6  (4.0)(5.0) | **645.3353** | **2** | **71.04** | **41.38** | **159** | - |
| **3.13** | 225/228 | 3.13-a-HyP 225/228 | **SLPLSP(+15.99)YSP(+15.99)QPGQEDR** | 146.8  (90.0)(61.3) | 81.3  (8.5)(10.4) | **901.9295** | **2** | **85.29** | **42.25** | **98** | P6:HyP:47.36;  P9:HyP:18.53 |
|  | 225 | 3.13-a-HyP 225 | SLPLSP(+15.99)YSPQPGQEDR | 39.9  (17.3)(43.3) | 15.7  (7.0)(44.7) | **893.9331** | **2** | **73.02** | **43.17** | **125** | P6:HyP:26.52 |
|  | - | 3.13-a | SLPLSPYSPQPGQEDR | 8.8  (5.5)(62.5) | 3.0  (2.3)(79.0) | **885.9360** | **2** | **79.02** | **45.21** | **72** | - |
|  | 239 | 3.13-b-HyP 239 | **EFSP(+15.99)QGQHGR** | 8.4  (3.2)(38.2) | 94.1  (4.5)(4.8) | **579.7675** | **2** | **49.85** | **15.20** | **61** | P4:HyP:1000.00 |
|  | - | 3.13-b | EFSPQGQHGR | 0.8  (0.4)(48.8) | 5.9  (4.5)(75.6) | **571.7700** | **2** | **38.89** | **23.18** | **24** | - |
|  | 525 | 3.13-c-HyP 525 | **FFVPPFQQSP(+15.99)R** | 137.3  (56.8)(41.4) | 32.6  (11.2)(34.3) | **455.9031** | **3** | **69.54** | **49.37** | **120** | P10:HyP:61.26 |
|  | - | 3.13-c | FFVPPFQQSPR | 234  (137.0)(58.4) | 67.4  (11.2)(16.6) | **675.3513** | **2** | **74.70** | **50.11** | **130** | - |
| **3.17/20** | 222/225 | 3.17/20-a-HyP 222/225 | **SLPYSP(+15.99)YSP(+15.99)QTQPK** | 316.4  (66.2)(20.9)(34.6) | 79.0  (9.4)(11.9) | **542.2645** | **3** | **60.59** | **37.45** | **90** | P6:HyP:47.09;  P9:HyP:34.30 |
|  | 222 | 3.17/20-a-HyP 222 | SLPYSP(+15.99)YSPQTQPK | 5.5  (3.8)(69.2)(60.6) | 0.8  (0.9)(119.8) | **803.8975** | **2** | **64.67** | **41.15** | **163** | P6:HyP:34.94 |
|  | 225 | 3.17/20-a-HyP 225 | SLPYSPYSP(+15.99)QTQPK | 61.8  (31.4)(50.9)(28.2) | 15.0  (5.7)(38.2) | **536.93** | **3** | **47.33** | **41.57** | **16** | P9:HyP:28.74 |
|  | - | 3.17/20-a | SLPYSPYSPQTQPK | 22.5  (17.4)(77.5)(36.3) | 5.2  (3.5)(67.5) | **796.9006** | **2** | **59.40** | **42.94** | **115** | - |

Table of HyP-modified peptides with a mean abundance ≥ 2 fmoles on column or ≥ 5 % % (w/w) detected and an Ascores >10 are shown. Allergen family, isoforms in family detected by these peptides are also listed. Tryptic peptide sequence used (with oxidation modification indicated: +15.99), m/z and z of peptide and retention time (RT) are also shown. -10LogP probability scores reported by PEAKS v8.5 indicates the scoring significance of a peptide-spectrum match. Ascore: Localization score assigned to modifications on the peptide as reported by PEAKS v8.5

Bolded peptides indicate the most abundant peptide. Each peptide is named by allergen isoform, peptide site of hydroxylation (e.g a, b…) and is discriminated by the detected site of HyP as indicated by the associated amino acid number. Unmodified peptides are reported without the associated amino acid number. ^1^ Mean Abundance: Defined as detected peptide fmol on column (after Gly Phos normalization) of the 20 genotypes. ^2^ St Dev of abundance: Defined as the standard deviation of the mean abundance of the 20 genotypes. ^3^ CV of abundance: Defined as the standard deviation/mean of the Av abundance x 100 of the 20 genotypes (Variability of detection across the genotypes). ^4^ Mean percent detected: Defined as the percent abundance detected of each HyP or Non-HyP peptide versus the sum of all peptides from similar site of the 20 genotypes. ^5^ St Dev of mean percent: Defined as the standard deviation of the percent abundance detected of each HyP or Non-HyP peptide versus the sum of all peptides from similar site of the 20 genotypes. ^6^CV of percent: Defined as the standard deviation/mean of percent detected x 100 of the 20 genotypes


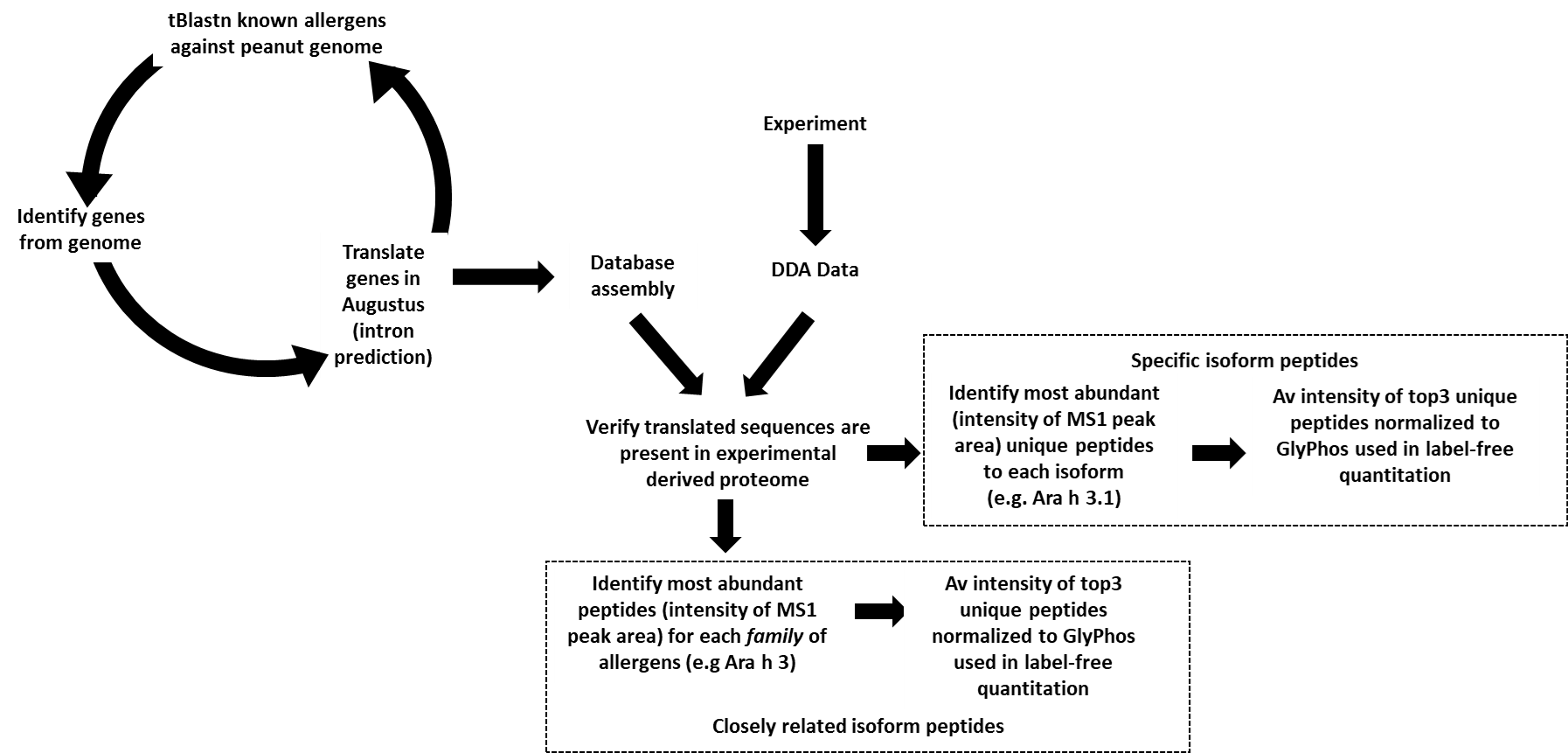


# Figure S1: Overview of experimental methodology

Generation of the Genomic Derived Peanut Seed Proteome and Peptide Identification Method


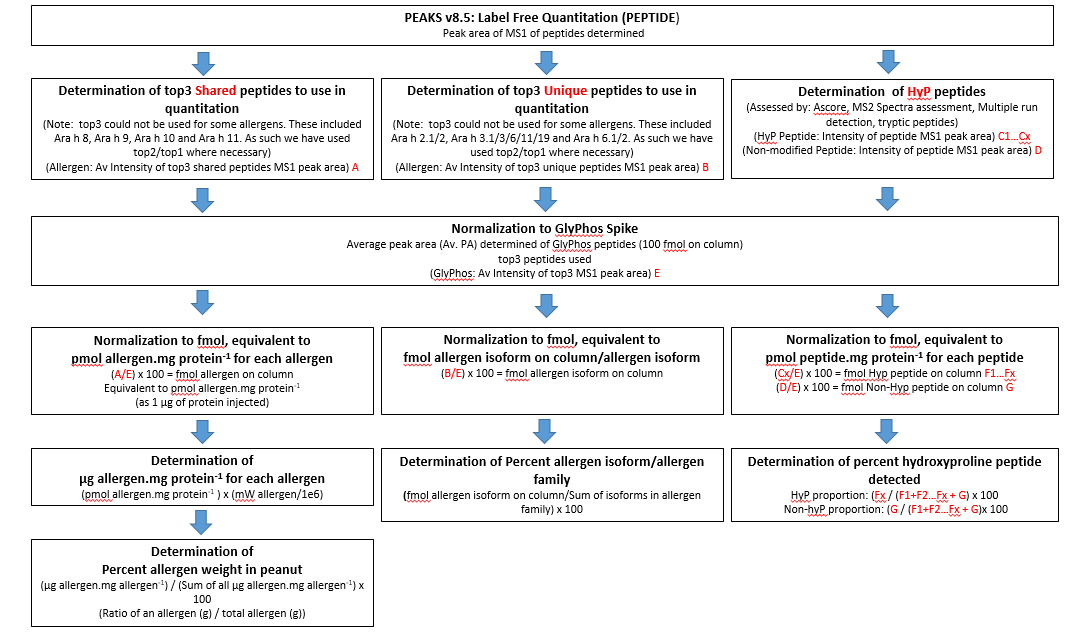


# Figure S2: Overview of data analysis methodology

A: Mean intensity of the top3 (peak area of MS1) shared peptides for each allergen (see excel supplementary)

B Mean intensity of the top3 (peak area of MS1) unique peptides for each allergen isoform (see excel supplementary)

Cx: Mean intensity (peak area of MS1) of each HyP-modified peptide for each HyP site (see excel supplementary)

D Mean intensity (peak area of MS1) of each unmodified peptide for each HyP site (see excel supplementary)

E Mean intensity of the top3 (peak area of MS1) peptides of glycogen phosphorylase spike

Fx: Normalized concentration of each HyP-modified peptide

G: Normalized concentration of the unmodified peptide for each HyP site





# Figure S3: Protein quantitation of Ara h 7, 8, 9, 10 and 11 as determined by closely related isoform (shared) peptide quantitation.

Allergen quantitation is expressed as protein content % (Weight/Weight). Each measurement was from 4 MS runs. Mean and standard error of the mean shown





# Figure S4: Comparison of market types within allergen families

Allergen quantitation is expressed as the protein content % (Weight (allergen)/Weight (total allergen)) p per market type and allergen family. Each measurement was from two technical replicates from each peanut genotype and includes the mean, percent coefficient of variation (CV%), maximum, and minimum values with respect to each allergen and market type (Runner, Spanish, Valencia, Virginia, Virginia Sub Group 1 (vg6, vg8, vg10 and vg11) and Virginia Sub Group 2 (vg1, vg2, vg3, vg4, vg5, vg7 and vg9). Market types share letters if they are not significantly different (two-way ANOVA and post-hoc Tukey’s test (p<0.001)). R: Runner; S: Spanish; V: Valencia and Vg: Virginia.





# **Figure S5: Isoform distribution of Ara h 1, Ara h 2 and Ara h 6, in all genotypes as determined by specific isoform (unique) peptide quantitation**.

Mean and standard error of the mean shown

# Figure S6: Examples of HyP modified and non-modified spectra

| **Ara h 1.1** | | | |
| --- | --- | --- | --- |
| WGP(+15.99)AEPR | WGPAEPR | EGEQEWGTP(+15.99)GSEVR | EGEQEWGTPGSEVR |
| 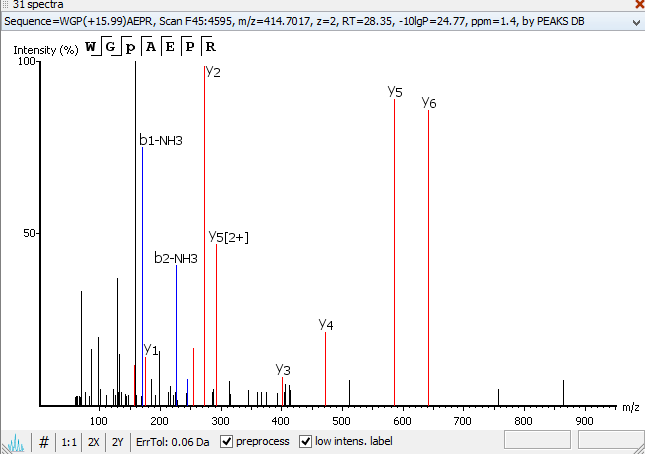 | 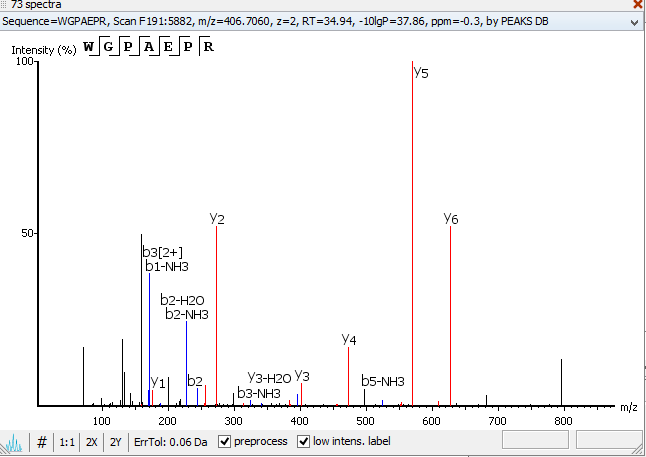 | 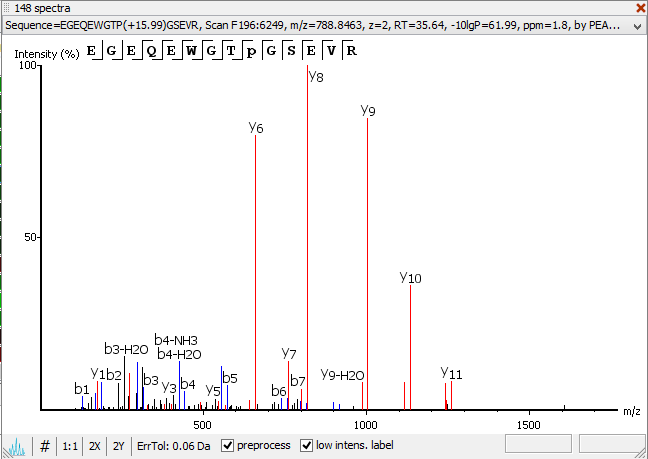 | 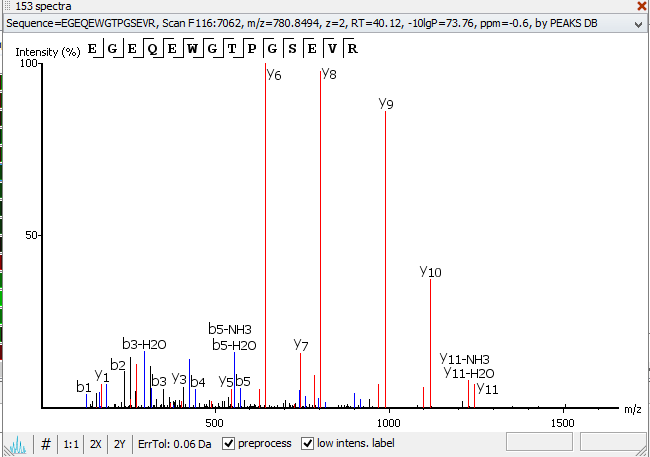 |
| **Ara h 1.2** | | | |
| ESHFVSARPQSQSQSP(+15.99)SSP(+15.99)EK | ESHFVSARPQSQSQSPSSPEK |  |  |
| 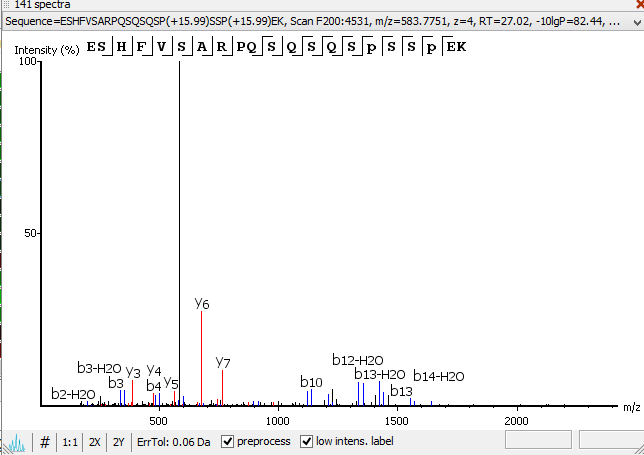 | ND |  |  |
| **Ara h 2.1** | | | |
| DPYSP(+15.99)SQDPYSP(+15.99)SP(+15.99)YDR | DPYSP(+15.99)SQDPYSP(+15.99)SPYDR | DPYSPSQDPYSP(+15.99)SPYDR | DPYSPSQDPYSPSPYDR |
| 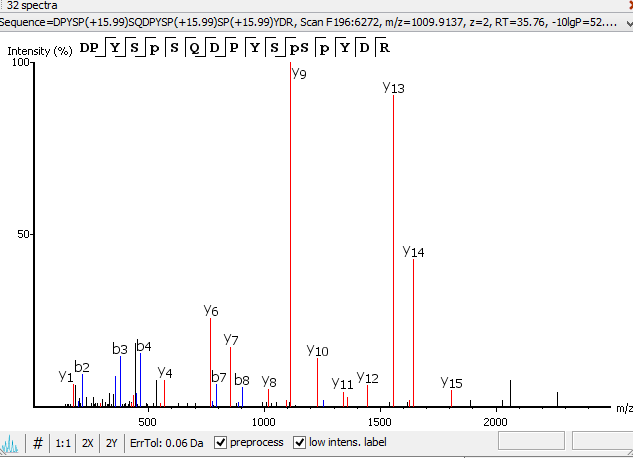 | 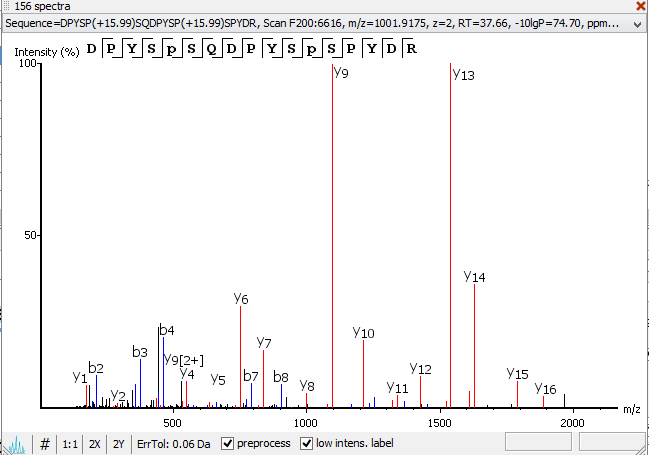 | 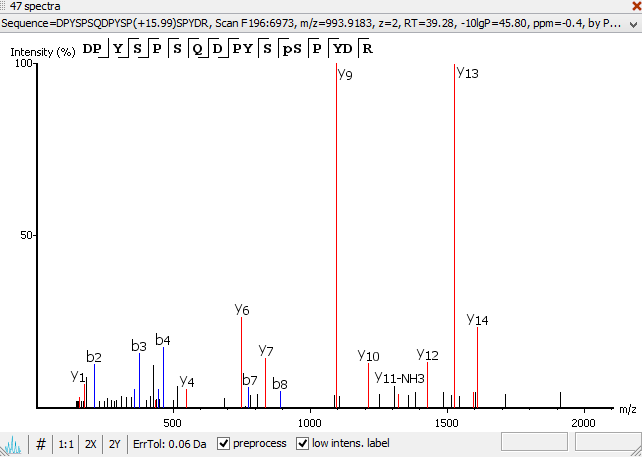 | ND |
| **Ara h 2.2** | | | |
| DPYSP(+15.99)SQDPYSP(+15.99)SQDPDR | DPYSPSQDPYSPSQDPDR | DPYSP(+15.99)SP(+15.99)YDR | DPYSP(+15.99)SPYDR |
| 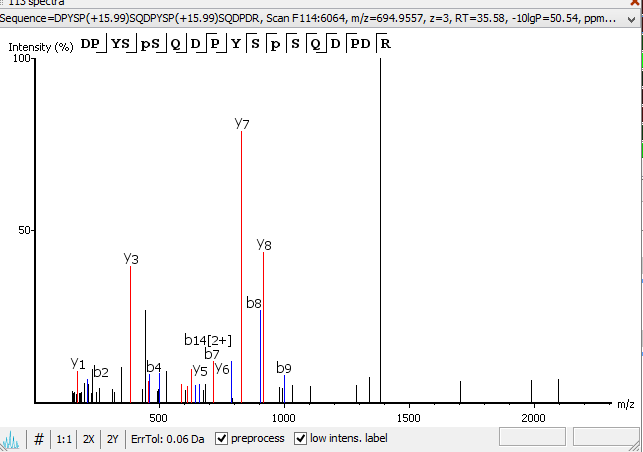 | ND | 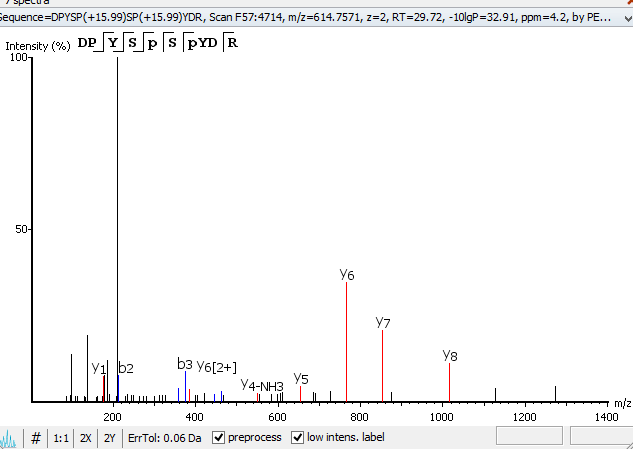 | 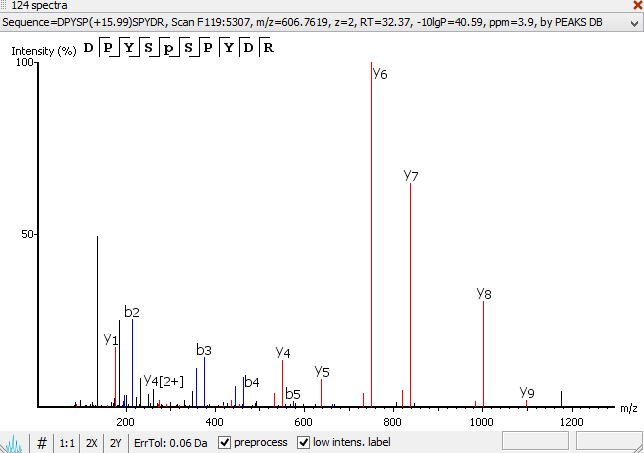 |
| DPYSPSPYDR |  |  |  |
| 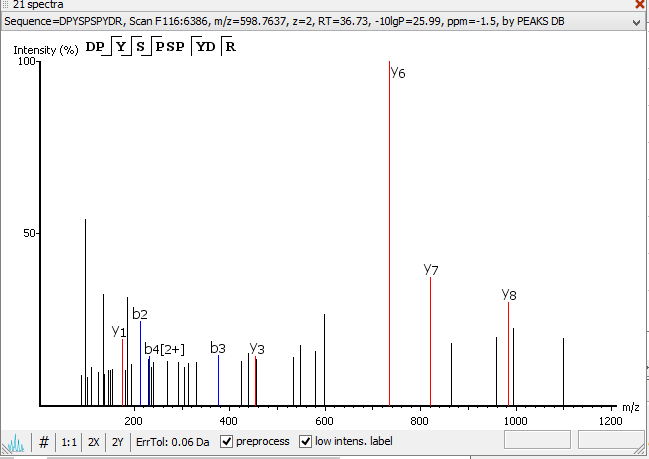 |  |  |  |
| **Ara h 3.4/5/10** | | | |
| SLPYSP(+15.99)YSP(+15.99)QSQPR | SLPYSPYSP(+15.99)QSQPR | SLPYSPYSPQSQPR |  |
| 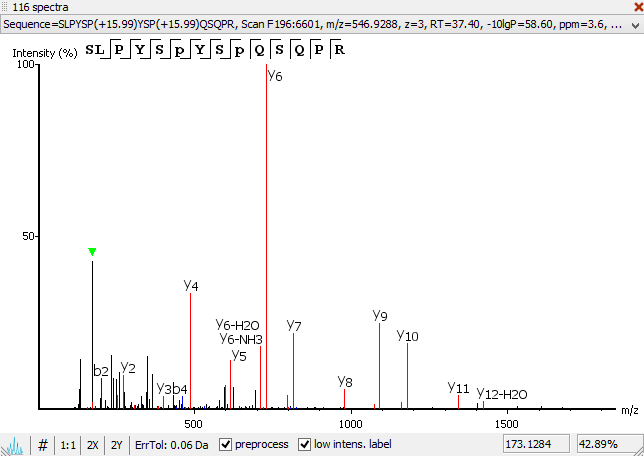 | 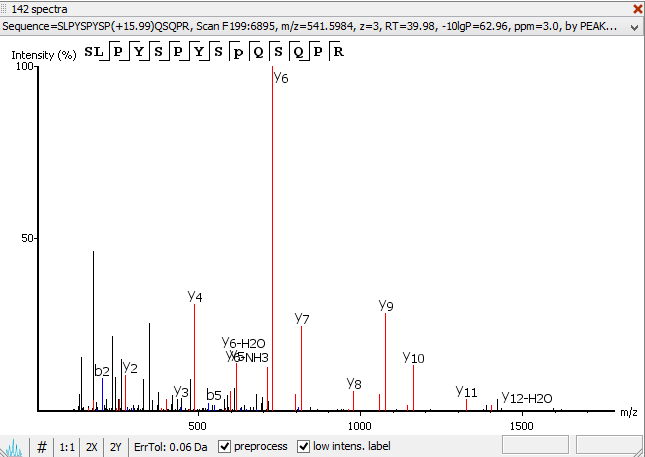 | 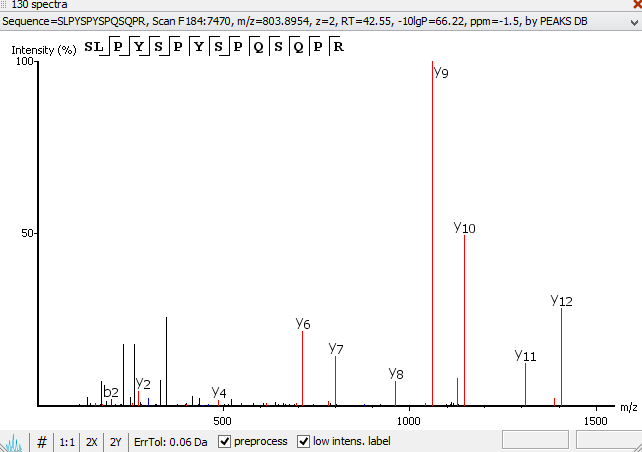 |  |
| **Ara h 3.10** | | | |
| FFVPPSQQSP(+15.99)R | FFVPPSQQSPR |  |  |
| 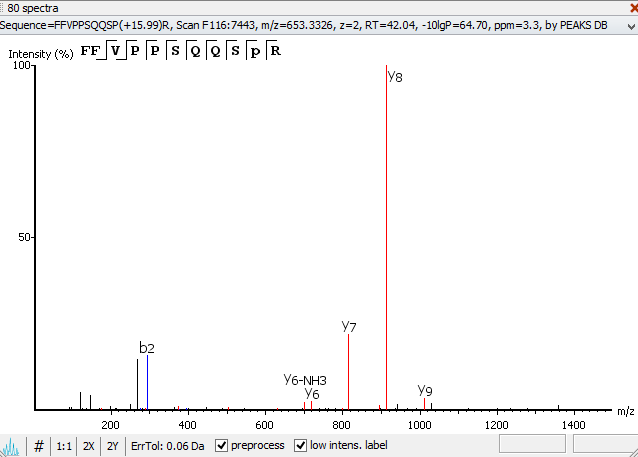 | 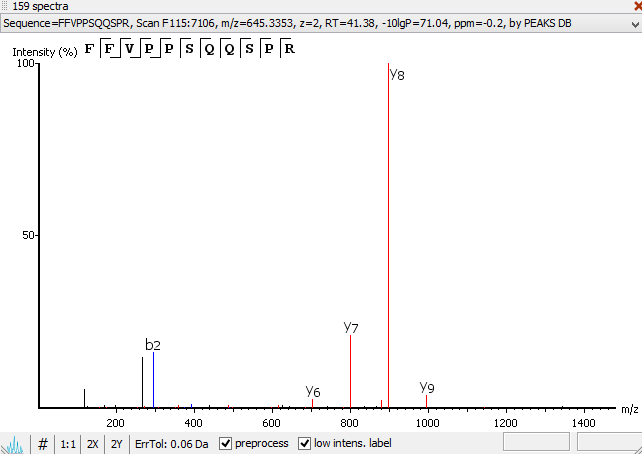 |  |  |
| **Ara h 3.13** | | | |
| SLPLSP(+15.99)YSP(+15.99)QPGQEDR | SLPLSP(+15.99)YSPQPGQEDR | SLPLSPYSPQPGQEDR |  |
| 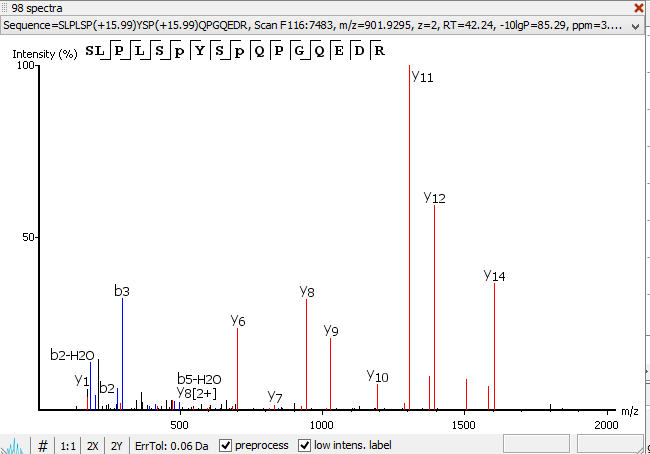 | 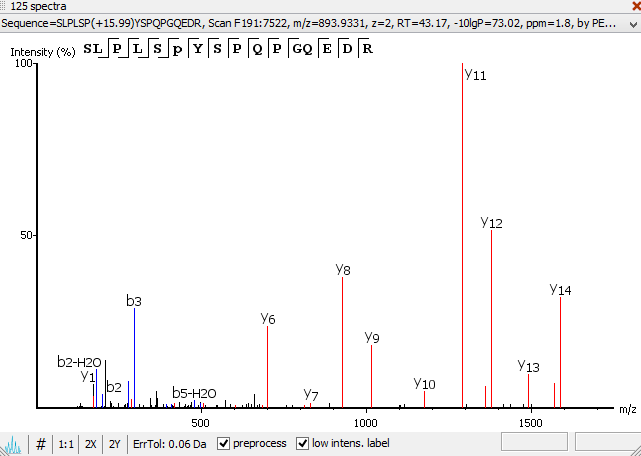 | 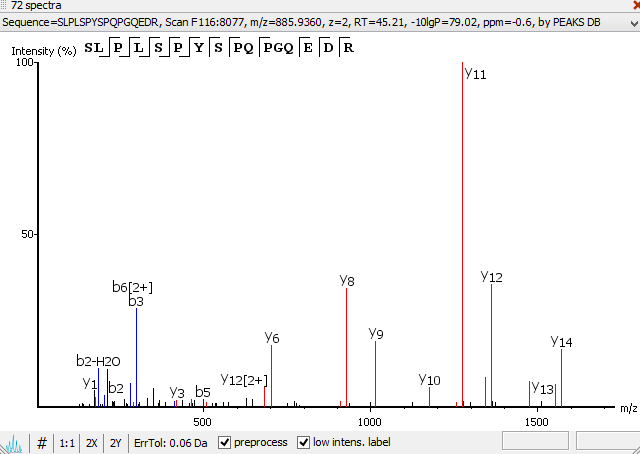 |  |
| EFSP(+15.99)QGQHGR | EFSPQGQHGR | FFVPPFQQSP(+15.99)R | FFVPPFQQSPR |
| 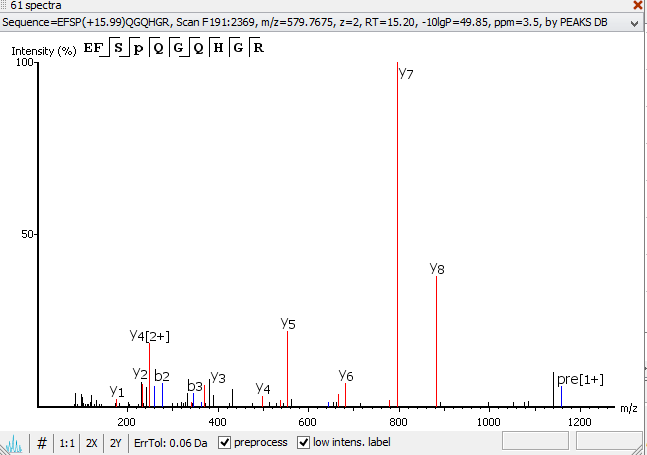 | 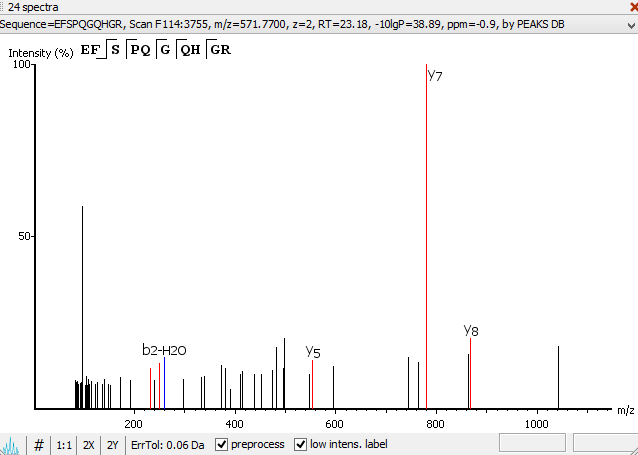 | 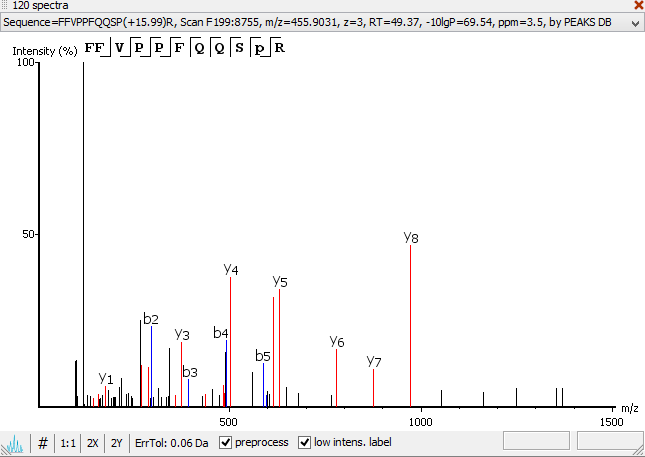 | 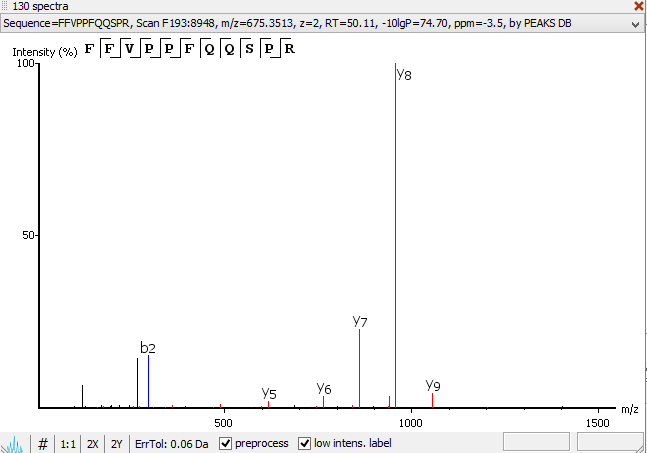 |
| **Ara h 3.17/20** | | | |
| SLPYSP(+15.99)YSP(+15.99)QTQPK | SLPYSP(+15.99)YSPQTQPK | SLPYSPYSP(+15.99)QTQPK | SLPYSPYSPQTQPK |
| 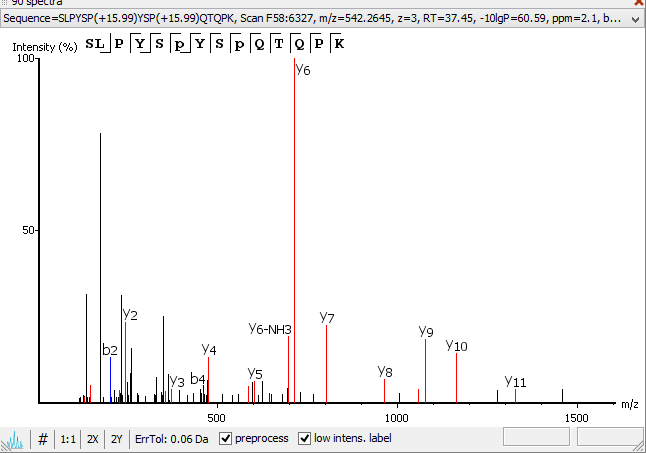 | 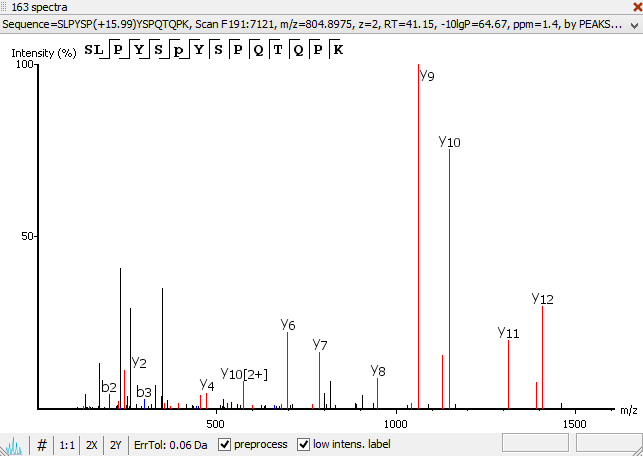 | 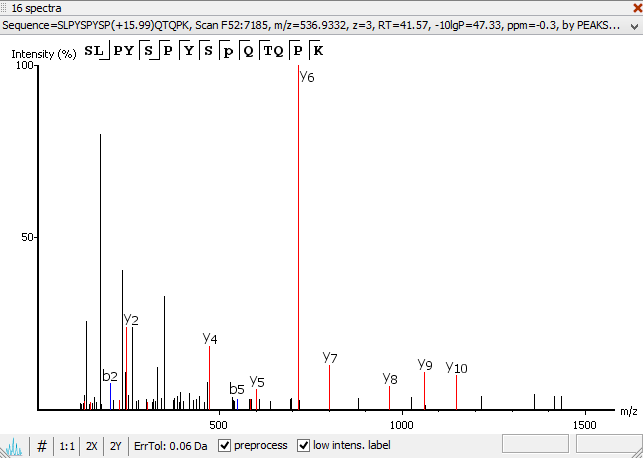 | 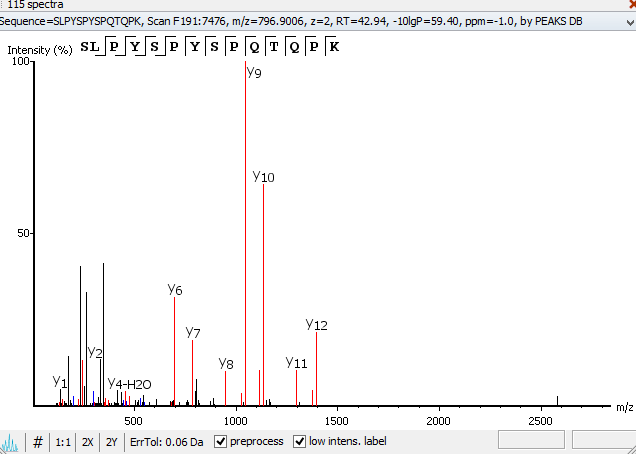 |





# Figure S7a-c: Stacked bar histogram plots show the average peptide quantitation of the hydroxyproline-rich sites in the Ara h 1 isoforms.

Determined by calculating the proportion of each peptide detected showing mean and standard error of the mean from two biological replicates, which had two technical replicates for each sample (left y-axis). Each peanut genotype analyzed is shown on the x-axis.





# Figure S8a-c: Stacked bar histogram plots show the average peptide quantitation of the hydroxyproline-rich sites in the Ara h 2 isoforms.

Determined by calculating the proportion of each peptide detected showing mean and standard error of the mean from two biological replicates, which had two technical replicates for each sample (left y-axis). Each peanut genotype analyzed is shown on the x-axis.








# Figure S9a-f: Stacked bar histogram plots show the average peptide quantitation of the hydroxyproline-rich sites in the Ara h 3 isoforms.

Determined by calculating the proportion of each peptide detected showing mean and standard error of the mean from two biological replicates, which had two technical replicates for each sample (left y-axis). Each peanut genotype analyzed is shown on the x-axis.


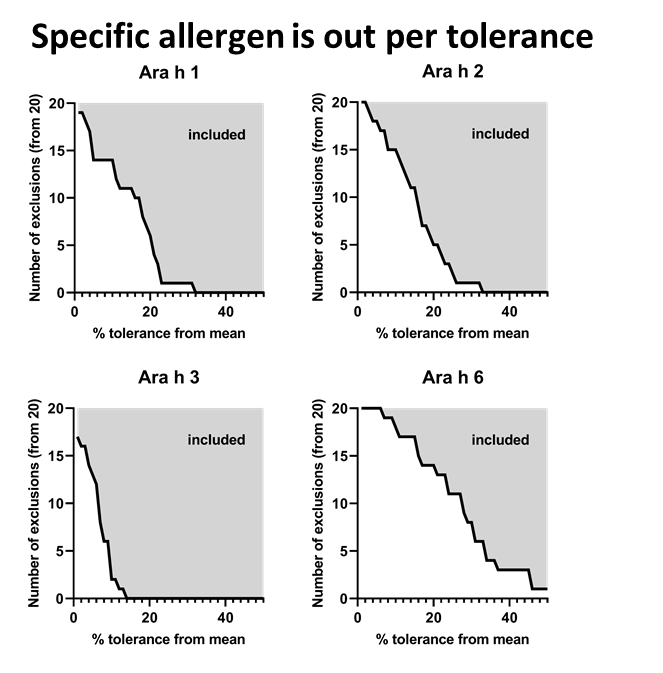


# Figure S10: Hypothetical tolerance criteria compared to the variance observed from the 20 peanut genotypes studied per allergen.

For each of the 20 genotypes studied, the quantities of Ara h 1, 2, 3 and 6 obtained by Shared peptide quantitation (percentage peanut protein (Allergen (g)/Total protein (g)) were compared to the overall mean per allergen family and screened as if they were clinical materials. Genotypes were separated into included or excluded groups per percentage tolerances from 0-50% given the relative difference of each of Ara h 1, 2, 3 and 6 to the overall mean per family as well as if any of these allergens were outside of a given tolerance level.
